# Design, Synthesis, and Mechanistic Anticancer Evaluation of New Pyrimidine-Tethered Compounds

**DOI:** 10.3390/ph18020270

**Published:** 2025-02-19

**Authors:** Farida Reymova, Belgin Sever, Edanur Topalan, Canan Sevimli-Gur, Mustafa Can, Amaç Fatih Tuyun, Faika Başoğlu, Abdulilah Ece, Masami Otsuka, Mikako Fujita, Hasan Demirci, Halilibrahim Ciftci

**Affiliations:** 1 Department of Bioengineering Sciences, Izmir Katip Celebi University, Izmir 35620, Türkiye; feridereymova@gmail.com (F.R.); mustafa.can@ikc.edu.tr (M.C.); 2 Department of Pharmaceutical Chemistry, Faculty of Pharmacy, Anadolu University, Eskisehir 26470, Türkiye; belginsever@anadolu.edu.tr; 3 Medicinal and Biological Chemistry Science Farm Joint Research Laboratory, Faculty of Life Sciences, Kumamoto University, Kumamoto 862-0973, Japan; motsuka@gpo.kumamoto-u.ac.jp (M.O.); mfujita@kumamoto-u.ac.jp (M.F.); 4 Department of Molecular Biology and Genetics, Koc University, Istanbul 34450, Türkiye; etopalan23@ku.edu.tr (E.T.); hdemirci@ku.edu.tr (H.D.); 5 Department of Basic Pharmaceutical Sciences, Faculty of Pharmacy, Izmir Katip Celebi University, Izmir 35620, Türkiye; canansevimli@yahoo.com; 6 Department of Chemistry, Faculty of Science, Istanbul University, Fatih, Istanbul 34126, Türkiye; aftuyun@istanbul.edu.tr; 7 Department of Pharmaceutical Chemistry, Faculty of Pharmacy, European University of Lefke, Northern Cyprus, TR-10, Mersin 99800, Türkiye; fabasoglu@eul.edu.tr; 8 Department of Medical Biochemistry, Faculty of Medicine, Biruni University, İstanbul 34015, Türkiye; aece@biruni.edu.tr; 9 Department of Drug Discovery, Science Farm Ltd., Kumamoto 862-0976, Japan; 10 Department of Molecular Biology and Genetics, Burdur Mehmet Akif Ersoy University, Istiklal Campus, Burdur 15030, Türkiye

**Keywords:** pyrimidine, chalcone, pyrazoline, thiazole, lung cancer, breast cancer, apoptosis, EGFR, induced fit docking, molecular dynamics simulation

## Abstract

**Background:** Despite recent breakthroughs in cancer treatment, non-small cell lung cancer (NSCLC) and breast cancer remain major causes of death from all malignancies. The epidermal growth factor receptor (EGFR) is an important mediator of the pathways involved in cell proliferation, apoptosis, and angiogenesis. Thus, its overexpression triggers several types of cancer, including NSCLC and breast cancer. **Methods:** In the current study, we synthesized new pyrimidine-tethered compounds (chalcone derivative (**B-4**), pyrazoline–carbothioamide (**B-9**), and pyrazoline–thiazole hybrids (**BH1-7**)). These compounds were then tested for cytotoxicity against A549 NSCLC and MCF-7 breast cancer cells. **Results:** Of these, **B-4** displayed significant cytotoxicity against both cells (IC_50_ = 6.70 ± 1.02 µM for MCF-7; IC_50_ = 20.49 ± 2.7 µM for A549) compared to the standard agent lapatinib (IC_50_ = 9.71 ± 1.12 µM for MCF-7; IC_50_ = 18.21 ± 3.25 µM for A549). The anticancer potential of **B-4** between Jurkat leukemic T cells and peripheral blood mononuclear cells (PBMCs) (healthy) was found to be selective. Mechanistically, 11.9% and 10.2% of A549 and MCF-7 cells treated with **B-4**, respectively, underwent apoptosis and **B-4** produced 46% EGFR inhibition at a concentration of 10 μM. The **B-4**/EGFR complex obtained after induced fit docking was subjected to 300 ns of molecular dynamics simulation, which confirmed the stability of the complex in a mimicked biological environment. On the other hand, **B-4** was shown to have drug-like properties by in silico pharmacokinetic estimation. **Conclusions: B-4** is an EGFR inhibitor and apoptosis inducer for future NSCLC and breast cancer studies.

## 1. Introduction

Cancer is the second leading global killer after cardiovascular disorders and the leading cause of death in people under the age of 85. The coronavirus disease 2019 (COVID-19) pandemic affected the prognosis of cancer for a while, delaying diagnosis and treatment of cancer due to hospital-acquired infections, comorbidities, social and economic damage, limiting the use of health care settings, and health insurance [1,2]. The World Health Organisation’s (WHO) cancer agency, the International Agency for Research on Cancer (IARC), has estimated the global burden of cancer. According to the report, lung cancer accounts for 2.5 million new cancer diagnoses, followed by female breast cancer at 2.3 million. Lung cancer is also the leading cause of cancer death with 1.8 million deaths, while breast cancer is responsible for 670,000 deaths, the highest number after lung, colorectal, and liver cancer types [3].

Tobacco use represents the most significant global risk factor for lung cancer [4], whereas environmental exposures, including the use of biomass fuels, arsenic, radon, industrial carcinogens, and air pollution, also exert a considerable influence on the incidence and mortality of lung cancer, with notable regional variations. The existence of disparate lung cancer subtypes and somatic mutations (e.g., epidermal growth factor receptor (EGFR) alterations) is indicative of geographic disparities in smoking patterns, environmental exposures, and genetics [5]. Furthermore, certain groups, including women, individuals infected with the human immunodeficiency virus (HIV), and never-smokers, exhibit distinctive characteristics [6,7,8]. Lung cancer is typically classified based on the origin of the cells and is broadly divided into non-small cell lung cancers (NSCLCs) and small cell lung cancers (SCLCs) [9]. NSCLC represents 80–90% of all lung cancer cases, with approximately 50–60% of patients being diagnosed at advanced or metastatic stages [10,11,12]. The survival rate for NSCLC remains low, with a 5-year survival rate of just 18% [11].

On the other hand, while breast cancer predominantly affects women, approximately 1% of cases occur in men [13]. Risk factors in developed countries include lifestyle changes, late childbirth, late-night work, and hormone replacement therapy, while in developing countries, high incidence and mortality are mainly due to lack of awareness, inadequate screening, delayed diagnosis, and limited medical resources [14,15,16,17]. Breast cancer can be categorized in a number of ways, but the most widely used classification divides it into four molecular subtypes: luminal A, luminal B, human epidermal growth factor receptor 2 (HER2)-positive, and triple-negative breast cancer (TNBC) [18]. Breast cancers that express no estrogen receptor (ER), progesterone receptor (PR), or HER2 are classified as TNBC. This category of breast cancer represents 10–20% of all cases [19,20].

The EGFR is a transmembrane receptor tyrosine kinase encoded by the EGFR gene, regulating essential cellular processes, including cell growth, metabolism, programmed cell death, and survival. The overexpression of the EGFR, gene amplification, and activating mutations are commonly observed in both NSCLC and certain subtypes of breast cancer. These genetic alterations contribute to tumor growth and resistance to therapy [5,21]. In NSCLC, this has established the EGFR tyrosine kinase (TK) as a critical target, prompting the development of various tyrosine kinase inhibitors (EGFR-TKIs) to suppress its abnormal activity [22]. At present, a number of EGFR-TKIs, including erlotinib, gefitinib, afatinib, and osimertinib, are available and are being actively employed in clinical practice to treat NSCLC, with a particular focus on targeting EGFR mutations [23]. Similarly, EGFR overexpression has been identified as a contributing factor in the development of HER2-positive and TNBC, where EGFR-targeted therapies, such as lapatinib and neratinib, have demonstrated considerable promise [24,25,26,27]. Upon binding to ligands such as the epidermal growth factor (EGF), the EGFR forms homodimers or heterodimers with ErbB receptors, leading to the activation of its TK domains. This activation triggers autophosphorylation and initiates key signaling pathways, including the MAPK (RAS–RAF–MEK–ERK) and PI3K–AKT–mTOR pathways [28]. These signaling pathways have been identified as promoting tumor growth, survival, and resistance to cell death, thereby providing further evidence to support the role of the EGFR as a critical driver of cancer progression [29]. EGFR-TKIs, such as erlotinib and gefitinib, work by competitively binding to the ATP-binding site of the EGFR TK domain, thereby preventing ATP from binding and disrupting the activation of the pathway [30,31,32]. As a result, EGFR-TKIs have emerged as a key therapeutic strategy in both NSCLC and certain breast cancer subtypes.

**Figure 1:**
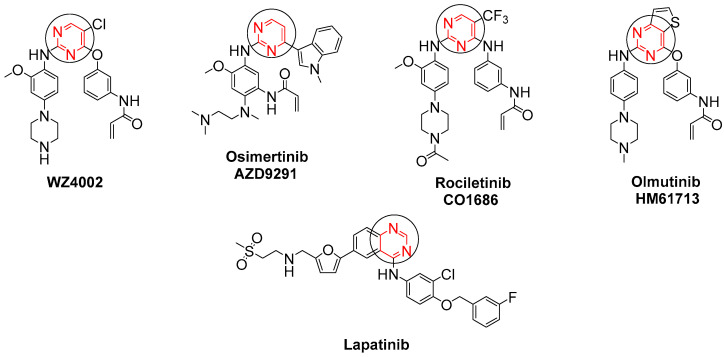
Pyrimidine-bearing EGFR inhibitors.

Since pyrimidine nucleotides are known to be essential components in the synthesis of deoxyribonucleic acid (DNA) and ribonucleic acid (RNA), pyrimidine-containing derivatives have been intensively investigated, especially for their potent antiproliferative effects [33]. So far, many pyrimidine-bearing analogs have been synthesized as EGFR inhibitors. Such inhibitors have been successfully designed to target different mutations in the TK domain of the EGFR and have been used in the treatment of cancers such as NSCLC. WZ40028 (Figure 1) is the first pyrimidine-carrying compound belonging to the third class of EGFR inhibitors to show promising inhibitory activity against mutant EGFR^T790M^. Osimertinib (AZD9291) (Figure 1) is the first pyrimidine-carrying drug active ingredient approved by the FDA, with very good clinical efficacy against NSCLC. In addition, rociletinib (CO1686) (Figure 1) and olmutinib (HM61713) (Figure 1) are other third class pyrimidine-based EGFR inhibitors. Due to the rapidly developing drug resistance that causes the long-term efficacy of these derivatives to decrease, studies for more effective pyrimidine-carrying EGFR inhibitors are ongoing [34]. Lapatinib (Figure 1), which is used as a standard agent in this study, is a small molecule belonging to the 4-anilinoquinazoline class (a bicyclic heterocycle with benzene fused to pyrimidine) [35].

**Figure 2:**
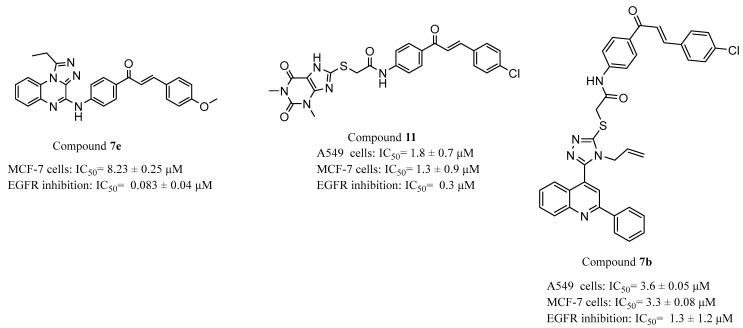
Chalcone derivatives, compounds **7e** [45], **11** [46], and **7b** [47], with EGFR inhibitory activity against NSCLC and/or breast cancer cells.

Chalcones are attracting considerable interest as potential anticancer candidates due to their relatively low side effects, high therapeutic index, and ease of synthesis. The 1,3-diphenyl-2-propen-1-one moiety can be easily modified to alter the biological potential of chalcones. Thus, chalcones have also been reported as having anticancer activity by inhibiting essential kinases such as the EGFR, vascular endothelial growth factor receptor-2 (VEGFR-2), and B-Raf (BRAF) kinase [36,37,38,39,40,41,42,43,44]. Anti-NSCL or anti-breast cancer activity associated with EGFR inhibition has been demonstrated for some of the chalcone derivatives (Figure 2) [45,46,47].

**Figure 3:**
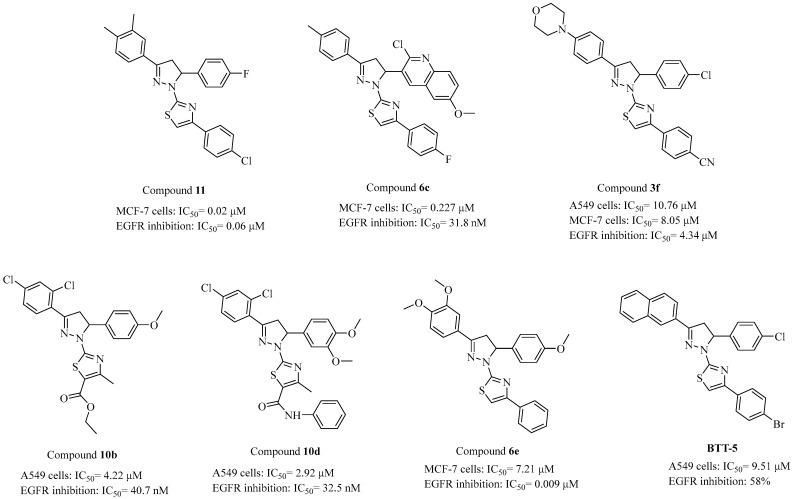
Thiazolyl–pyrazolines (compounds **11** [50], **6c** [51], **3f** [52], **10b** [53], **10d** [53], **6e** [54], and **BTT-5** [55]) with EGFR inhibitory activity against NSCLC and/or breast cancer cells.

Pyrazoline and thiazole are heterocyclic molecules with a wide range of biological profiles and a remarkable ability to influence the physico-chemical and biological properties of the compounds in which they are incorporated [48,49]. To date, pyrazoline–thiazole hybridization has been successfully used to design new conjugates with targeted anticancer activities, such as EGFR-directed activity against NSCLC and/or breast cancer (Figure 3) [50,51,52,53,54,55].

Based on the aforementioned data, in the current work, we synthesized new pyrimidine-based compounds (chalcone derivative (**B-4**), pyrazoline–carbothioamide (**B-9**), and pyrazoline–thiazole hybrids (**BH1-7**)). We then tested these compounds for their anticancer effects on A549 and MCF-7 cells, as well as their selective anticancer potential between Jurkat leukaemic T cells and peripheral blood mononuclear cells (PBMCs) (healthy). The most potent compound was further evaluated for its mechanistic anticancer effects, including inducing apoptosis in NSCLC and breast cancer cells and inhibiting the EGFR. Finally, for the most effective compound, molecular docking and molecular dynamic (MD) simulation were performed and several pharmacokinetic parameters were in silico anticipated.

## 2. Results

### 2.1. Chemistry

**Scheme 1:**
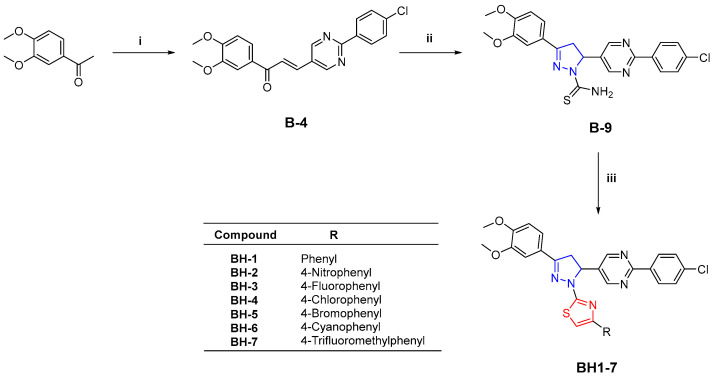
Reagents and conditions: (**i**) 2-(4-Chlorophenyl)pyrimidine-5-carbaldehyde, NaOH, ethanol, rt, 24 h. (**ii**) Thiosemicarbazide, NaOH, ethanol, reflux, 8–12 h. (**iii**) 2-Bromo-1-arylethanone, ethanol, reflux, 2–6 h.

In the current work, initially, (*E*)-3-(2-(4-chlorophenyl)pyrimidin-5-yl)-1-(3,4-dimethoxyphenyl)prop-2-en-1-one (**B-4**) was obtained via the Claisen Schmidt condensation of 1-(3,4-dimethoxyphenyl)ethan-1-one and 2-(4-chlorophenyl)pyrimidine-5-carbaldehyde under a basic condition. After that, 5-(2-(4-chlorophenyl)pyrimidin-5-yl)-3-(3,4-dimethoxyphenyl)-4,5-dihydro-1*H*-pyrazole-1-carbothioamide (**B-9**) was synthesized as a result of the reaction of **B-4** and thiosemicarbazide in the presence of NaOH. Finally, new pyrazoline–thiazole hybrids (**BH1-7**) were afforded through Hantzsch thiazole synthesis, the condensation of α-haloketones (2-bromo-1-arylethanones) and thioamides (**B-9**) (Scheme 1).

**Figure 4:**
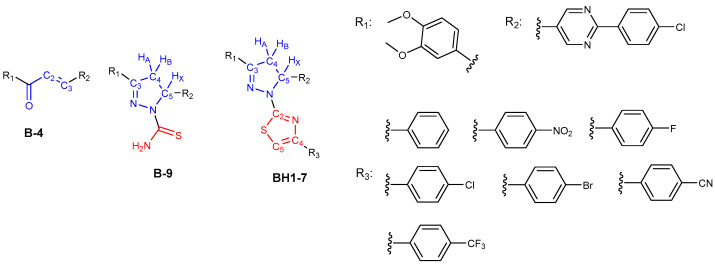
NMR diagram for **B-4**, **B-9**, and **BH1-7**.

The structures of **B-4**, **B-9**, and **BH1-7** were confirmed by spectral data (^1^H nuclear magnetic resonance, NMR), ^13^C NMR, and high-resolution mass spectra (HRMS). In the ^1^H NMR spectra of **B-4**, the characteristic propenone protons appeared as a multiplet at 7.69–7.74 ppm along with aromatic protons. In the ^13^C NMR spectra, the C_2_, C_3_, and carbonyl carbons of the propenone moiety were detected at 124.16 ppm, 144.89 ppm, and 187.48 ppm, respectively (Figure 4). The amine protons, which were observed as a singlet at 8.71 ppm, the characteristic H_A_ and H_X_ pyrazoline protons, resonating as doublets of doublets at 3.23 ppm (*J_AB_* = 17.55 Hz, *J_AX_* = 3.70 Hz) and 6.05 ppm (*J_AX_* = 3.70 Hz, *J_BX_* = 11.35 Hz), and the H_B_ proton seen as a multiplet at 3.83–3.92 ppm, confirmed the main chemical structure of **B-9**. In addition, the C_3_, C_4_, and C_5_ carbons of pyrazoline appeared at 152.35 ppm, 42.44 ppm, and 59.23 ppm, respectively. The C=S carbon, which appeared at 176.05 ppm, was also important in confirming the chemical structure of **B-9** (Figure 4). The condensation of the thiocarbamoyl of **B-9** to the thiazoles of **BH1-7** is evidenced by the appearance of the C_2_, C_4_, and C_5_ thiazole carbons of **BH1-7** at 164.09–165.42 ppm, 146.41–151.17 ppm, and 103.50–108.14 ppm, respectively. Moreover, the formation of **BH1-7** was enhanced by the presence of H_A_, H_B_, and H_X_ protons and C_3_, C_4_, and C_5_ carbons, corresponding to the pyrazoline pattern (Figure 4).

### 2.2. Cytotoxicity

**Figure 5:**
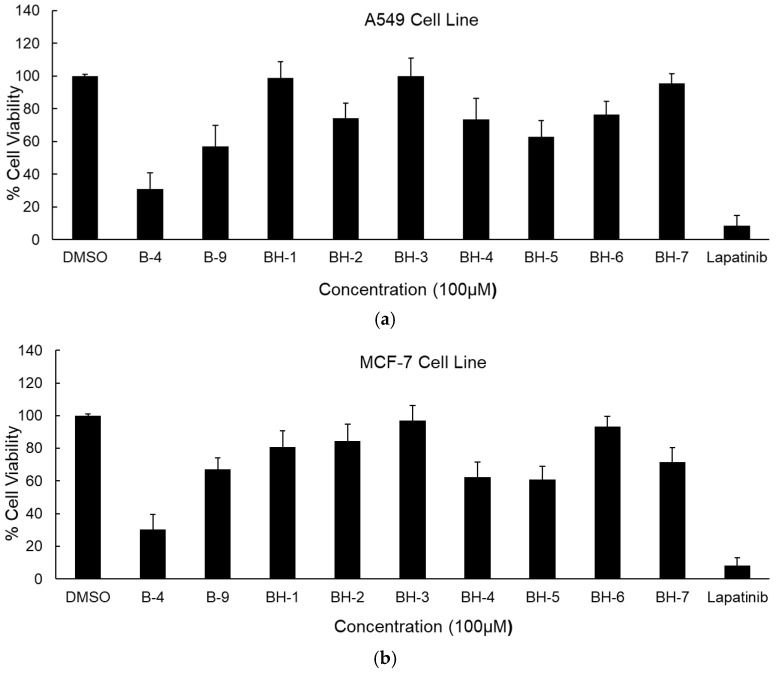
Cytotoxicity values of **B-4**, **B-9**, and **BH1-7** against A549 (**a**) and MCF-7 (**b**) cells at 100 µM concentration compared to lapatinib.

The new pyrazoline–thiazole hybrids (**BH1-7**) and new intermediates **B-4** and **B-9** were evaluated for cytotoxic activity against the A549 and MCF-7 cell lines compared to lapatinib using the MTT (3-(4,5-dimethyl-2-thiazolyl)-2,5-diphenyltetrazolium bromide) assay. The anticancer activity of **BH1-7**, **B-4**, and **B-9** against A549 and MCF-7 cells was initially screened in three replicates at a concentration of 100 µM (Figure 5a,b). Intriguingly, only the chalcone derivative **B-4** was found to reduce cell viability by more than 50% at a concentration of 100 µM against both cells. **B-4** (70%) was followed by **B-9** and **BH-5** at this concentration with 43% and 37% anti-NSCLC activity, respectively (Figure 5a). In addition, the anti-breast cancer activity of **B-4** (70%) was followed by that of **BH-5**, **BH-4**, and **B-9** with the values of 39%, 38%, and 33%, respectively (Figure 5b).

**B-4** was the only molecule with over 50% inhibition against both A549 and MCF-7 cells at 100 µM and was therefore tested at 5 doses (1, 3, 10, 30, and 100 µM) (three replicates at each concentration), and the IC_50_ values were calculated (Table 1). Compared to its anti-NSCLC activity, the anti-breast cancer activity of **B-4** was found to be higher. **B-4** displayed more significant anticancer activity against the MCF-7 cell line with an IC_50_ value of 6.70 ± 1.02 µM compared to lapatinib (IC_50_ = 9.71 ± 1.12 µM), while the anti-NSCLC activity profile of **B-4** (IC_50_ = 20.49 ± 2.71 µM) was similar to that of lapatinib (IC_50_ = 18.21 ± 3.25 µM) (Table 1).

As shown in Table 1, the selectivity of the anticancer activity of **B-4** was also examined between Jurkat T cells and PBMCs (healthy). The SI value found by the ratio of the IC_50_ value for Jurkat cells (1.50 ± 0.40 µM) to the IC_50_ value for PBMCs (68.89 ± 10.28 µM) was 45.93 for **B-4** compared to lapatinib (SI = 8.89).

### 2.3. Apoptosis

**Figure 6:**
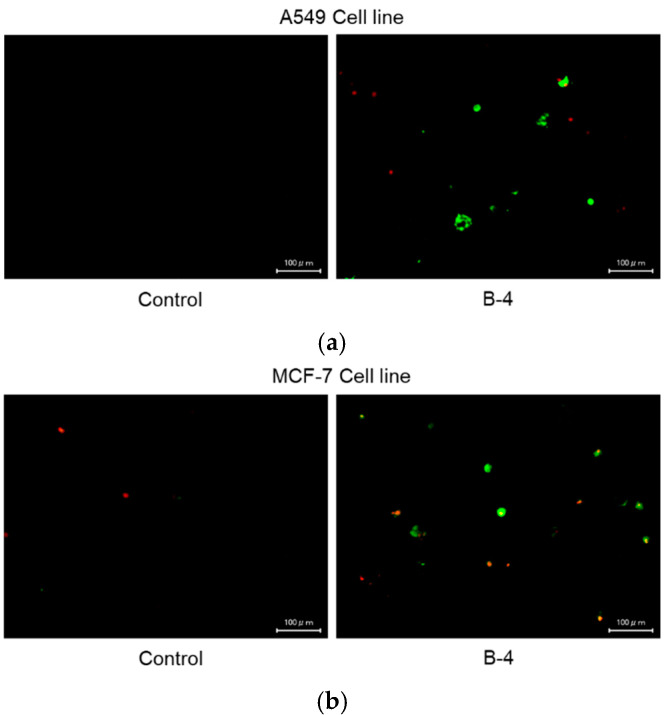
Changes in A549 (**a**) and MCF-7 (**b**) cells after control and **B-4** treatment for 24 h.

Since **B-4** was found to be the most effective and selective anticancer agent, the level of apoptosis in both A549 (Figure 6a) and MCF-7 (Figure 6b) cells was assessed by using the Annexin V/ethidium homodimer III staining technique, with green, yellow, and red staining indicating apoptotic, necrotic, or late apoptotic cells, respectively. This staining was observed by a fluorescence microscope. The findings showed that **B-4** induced apoptosis in 11.9% and 10.2% of A549 and MCF-7 cells, respectively.

### 2.4. EGFR Inhibition

**Figure 7:**
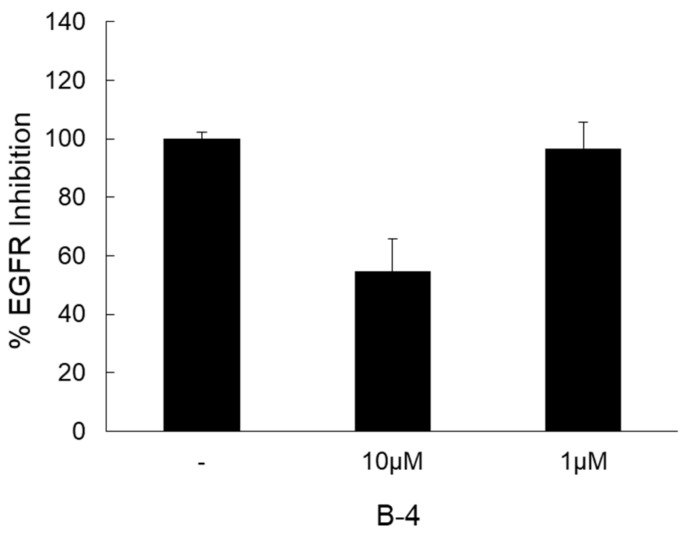
EGFR inhibition of **B-4** at 10 μM and 1 μM concentrations.

The EGFR is a key transmembrane protein in the occurrence and development of NSCLC and breast cancer. The strong anti-NSCLC and anti-breast cancer activity profile of **B-4** led us to investigate its ability to inhibit the EGFR. According to the results, **B-4** showed 46% EGFR inhibition at a concentration of 10 μM, with weak inhibition at a concentration of 1 μM (Figure 7).

### 2.5. Molecular Docking and MD Simulation

In order to evaluate the reliability of the molecular docking, as an internal validation [56,57,58], the native ligand in the X-ray crystal structure was first docked into the active pocket of the EGFR. The root mean square deviation (RMSD) between the resulting and the X-ray conformations was calculated as 0.97 Å. The 3D superimposed conformations of the native ligand and the docked pose are given in Supplementary Information (Figure S1).

**Figure 8:**
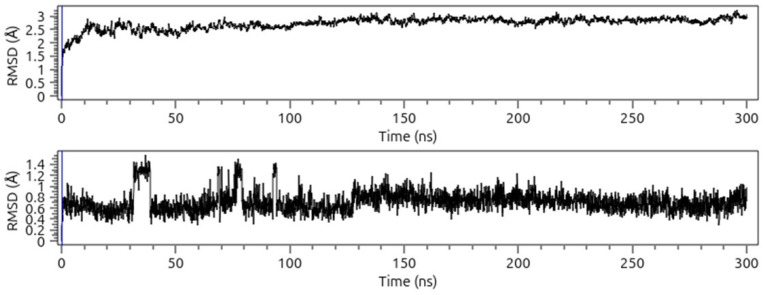
The calculated root mean square deviations of the EGFR (**top**) and the **B-4** (**bottom**) during 300 ns of MD run.

In general, molecular docking may not give reliable results as it does not consider a biological environment. Hence, we consulted MD simulations in which we put the **B-4**/EGFR docked pose in a sample of water and salt concentration and simulated it for 300 ns. Several metrics were calculated at the end of the simulation to evaluate the results. As can be clearly seen from Figure 8, the protein–ligand complex is quite stable during the whole simulation period. The RMSD of the protein reaches equilibrium after just ~10 ns and stays lower than 3 Å with very small variations. Besides staying in the binding site, the very low RMSD value of the ligand shows that compound **B-4** almost did not change its overall position.

**Figure 9:**
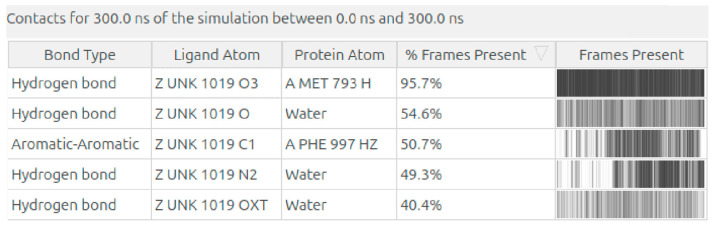
Frequency of the contacts generated after 300 ns of the MD simulation. Only contacts that are observed at least for 40% of the simulation time are shown.

The frequencies of the ligand contacts during 300 ns of the simulation were calculated (Figure 9) as well. During more than 95% of the entire simulation, a hydrogen bond between the carbonyl oxygen of **B-4** and the amino acid residue MET793 was observed. A π-π interaction between the 1,2-dimethoxybenzene ring of **B-4** and PHE997 was also observed for more than half of the MD simulation. The nitrogen atom in the pyrimidine ring and methoxy oxygens of **B-4** act as a hydrogen bond acceptor and engage in strong hydrogen bond interactions with the waters in the EGFR binding site.

**Figure 10:**
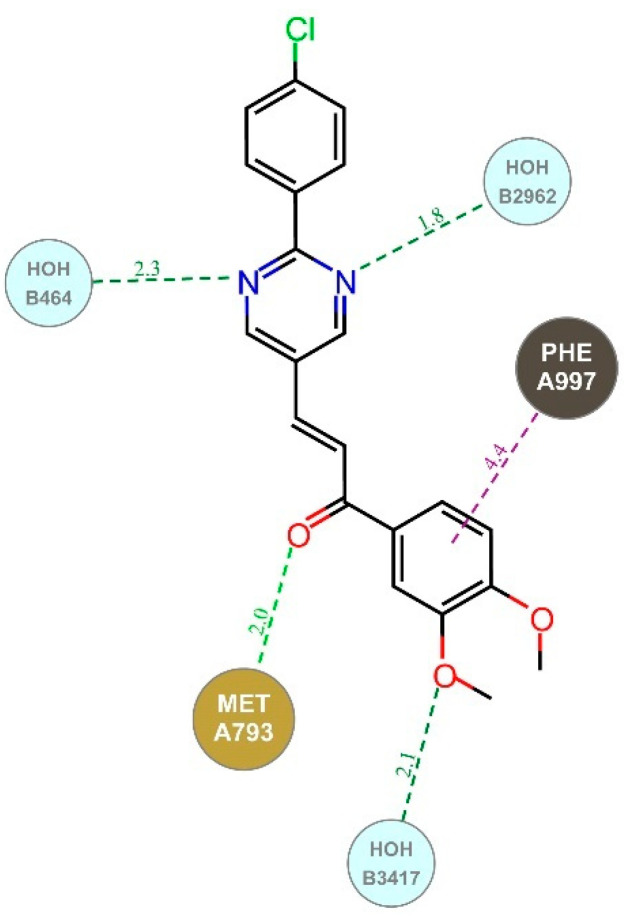
The interaction map for **B-4**/EGFR complex extracted from the most populated cluster during MD run.

We also carried out *k*-Means cluster analysis to group together all frames of the MD trajectories having similar protein conformations. Out of 10 generated clusters, we selected the most populated cluster, which contains the highest fraction of the overall frames. The 2D interaction map of **B-4**/EGFR is depicted in Figure 10. Notably, the 2D interaction map of the selected cluster is in well agreement with the frequency of the ligand contacts.

### 2.6. In Silico Pharmacokinetic Estimation

**B-4** was subjected to in silico profiling for various absorption, distribution, metabolism, and excretion (ADME) parameters such as the coefficients of octanol/water partition (QPlogPo/w), aqueous solubility (QPlogS), and human serum albumin binding (QPlogKhsa). The compatibility of **B-4** with Lipinski’s rule of 5 and Jorgensen’s rule of 3 was also determined using the QikProp module [59]. In addition, the in silico inhibition potential of **B-4** on various cytochrome P450 (CYP) enzymes including CYP1A2, CYP2C19, CYP2C9, CYP2D6, and CYP3A4, together with the assessment of blood–brain barrier (BBB) permeability, was implemented using the SwissADME web service [60].

**B-4** demonstrated a pronounced pharmacokinetic profile in which all metrics were within the appropriate intervals, as follows: QPlogPo/w, QPlogS, and QPlogKhsa were calculated with values of 4.451, −5.634, and 0.371, respectively, within the ranges (−2 to 6.5, −6.5 to 0.5, and −1.5 to 1.5, respectively). On a scale of 0–100% (>80% is high; <25% is low), **B-4** also displayed impressive oral absorption (100%) in humans. The parameters of Lipinski’s rule of 5 and Jorgensen’s rule of 3 were also fulfilled by **B-4**.

**Figure 11:**
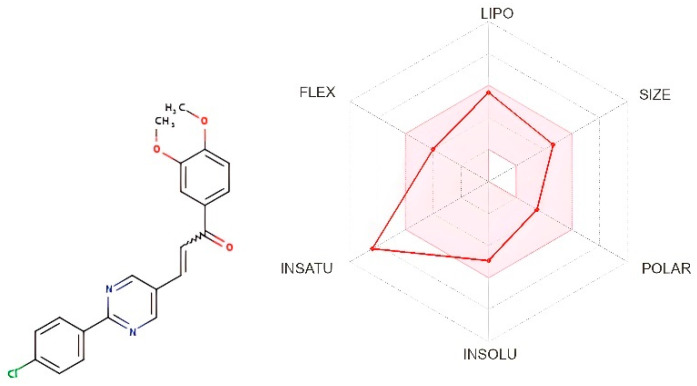
The chemical structure and bioavailability radar of **B-4** from the SwissADME webtool.

The values for oral bioavailability, including saturation (INSATU), size (SIZE), polarity (POLAR), solubility (INSOLU), lipophilicity (LIPO), and flexibility (FLEX), were shown in the pink area of the radar (Figure 11). **B-4** was found only outside the INSATU, while for the other values, it was in the pink area. **B-4** was associated with the inhibition of CYP1A2, CYP2C19, CYP2C9, and CYP3A4, but not CYP2D6. This suggests that **B-4** may cause potential drug–drug interactions.

**Figure 12:**
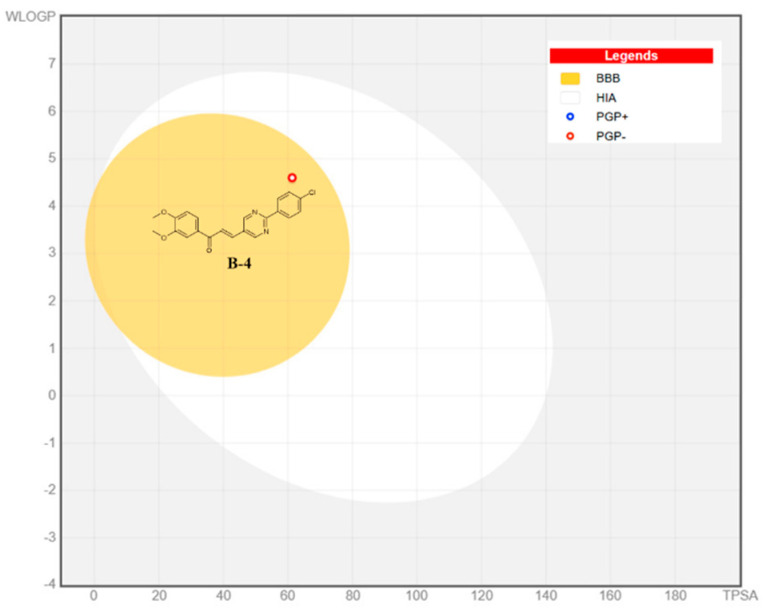
The chemical structure and bioavailability radar of **B-4** from the SwissADME webtool. BBB: Blood–brain barrier; HIA: human intestinal absorption; PGP: P-glycoprotein.

The BOILED egg model (Figure 12) describes whether a molecule has properties for the passive gastrointestinal absorption and penetration of the blood–brain barrier (BBB). The results showed that **B-4** was predicted to penetrate the brain (shown in yellow) and not act as a substrate for P-glycoprotein (red dot), reducing the possibility of its resistance to cancer cell lines by efflux.

## 3. Discussion

In the current work, we designed and synthesized new pyrimidine-based compounds (chalcone derivative (**B-4**), pyrazoline–carbothioamide (**B-9**), and pyrazoline–thiazole hybrids (**BH1-7**). We then evaluated the anticancer effects of these compounds against A549 NSCLC and MCF-7 breast cancer cells by MTT assay and, more mechanistically, the apoptotic effects in A549 and MCF-7 cells and the EGFR inhibitory potential of the most effective compound. In silico studies were also performed to determine the binding potential to the ATP binding site of the EGFR and key pharmacokinetic determinants of the most potent compound.

WZ40028, osimertinib, rociletinib (CO1686), and olmutinib (HM61713) are well-known pyrimidine-based EGFR inhibitors. In addition, the quinazoline ring, which is a fused bicyclic heterocycle with benzene and pyrimidine, is found in most EGFR inhibitors, such as lapatinib (Figure 1). Meanwhile, chalcone derivatives have been delved into for their potential EGFR-directed activities against NSCLC and/or breast cancer. Alshwah et al., 2017 [45] have reported the triazoloquinoxaline–chalcone derivatives for their EGFR-related anticancer effects, including anti-breast cancer activity. Compound **7e** (Figure 2) revealed anticancer activity against MCF-7 cells with an IC_50_ value of 8.23 ± 0.25 μM and EGFR inhibition (IC_50_ = 0.083 ± 0.04 μM). In another study, among xanthine/chalcone hybrids, compound **11** (Figure 2) showed cytotoxicity against A549 and MCF-7 cells with IC_50_ values of 1.8 ± 0.7 μM and 1.3 ± 0.9 μM, respectively. Compound **11** also inhibited the EGFR with an IC_50_ value of 0.3 μM [46]. Quinoline/chalcone hybrids with 1,2,4-triazole moiety were examined for EGFR-directed anti-NSCLC and anti-breast cancer activity. Compound **7b** (Figure 2) exhibited anticancer effects on A549 and MCF-7 cells with IC_50_ values of 3.6 ± 0.05 μM and 3.3 ± 0.08 μM, respectively. The EGFR inhibitory effect of compound **7b** was found as 1.3 ± 1.2 μM [47]. All these studies indicated that the anti-NSCLC and anti-breast cancer potential of chalcones was boosted by the combination of chalcones with various heterocycles bearing multiple azoles.

With regard to the studies involving the anti-NSCLC and anti-breast cancer activity of thiazolyl–pyrazoline hybrids associated with EGFR inhibition, there are promising results. Lv et al., 2011 [50] and George et al., 2019 [51] observed that compounds **11** (Figure 3) and **6c** (Figure 3) showed remarkable anticancer activity against the MCF-7 cell line with IC_50_ values of 0.07 μM and 0.227 μM, respectively. Compounds **11** and **6c** also inhibited the EGFR with IC_50_ values of 0.06 μM and 31.80 nM, respectively. In another study, we demonstrated that compound **3f** (Figure 3) was cytotoxic against both A549 NSCLC and MCF-7 cells with IC_50_ values of 10.76 μM and 8.05 μM, respectively. Compound **3f** was also able to inhibit the EGFR with an IC_50_ value of 4.34 μM [52]. Abdelsalam et al., 2022 [53] identified two compounds (compounds **3b** and **3d**) (Figure 3) in this series showing notable anti-NSCLC activity, inhibiting EGFR at nanomolar levels. On the other hand, Fakhry et al., 2022 [54] demonstrated that compound **6e** (Figure 3) revealed cytotoxicity towards MCF-7 cells and EGFR inhibitory activity with IC_50_ values of 7.21 μM and 0.009 µM, respectively. In our other study [55], compound **BTT-5** (Figure 3) was determined to possess anti-NSCLC (IC_50_ = 9.51 μM) activity and selective EGFR inhibitory (58.32%) effects at a 10 µM concentration. As shown in these studies, the methoxy and halogen substitutions on the phenyl ring had a positive effect on the anticancer activity of pyrazoline–thiazole hybrids.

In our current study, the chalcone derivative **B-4** was found to be the most effective cytotoxic and apoptotic agent against both A549 and MCF-7 cells. This indicates that the propenone (**B-4**) moiety improved the anticancer activity compared to pyrazoline–carbothioamide (**B-9**) and pyrazoline–thiazole (**BH1-7**) scaffolds. Among **BH1-7**, the 4-bromophenyl substitution increased the anti-NSCLC and anti-breast cancer activity, as shown in **BH-5**. This result is also consistent with our previous findings, as shown in **BTT-5** [55].

**B-4** also exhibited cytotoxic effects against Jurkat cells, showing less cytotoxicity against PBMCs (healthy). The SI value, which was calculated between the IC_50_ values of the PBMCs and the Jurkat cells, indicates a high degree of selectivity of **B-4**. The high anticancer activity of **B-4** against Jurkat cells also suggested that **B-4** might act on other kinases in addition to the EGFR.

**B-4**-treated A549 and MCF-7 cells underwent significant apoptosis after 24 h. In addition, **B-4** significantly inhibited the EGFR at a concentration of 10 µM. Molecular docking studies supported the importance of the propenone moiety for key hydrogen bonding in the ATP binding site of the EGFR.

The ADME analysis put emphasis on **B-4′**s drug-like properties of optimal lipophilicity, water solubility, and binding to human serum albumin, which might influence the volume of distribution and half-life of **B-4**. One of the main sites of metastasis in NSCLC is the brain. The ability of drugs to cross the BBB is therefore very important. **B-4** has been shown to be capable of BBB penetration. **B-4** has the potential to be metabolized by the CYP enzymes tested, with the exception of CYP2D6, resulting in possible drug–drug interactions.

## 4. Materials and Methods

### 4.1. Chemistry

All reagents have been purchased and used commercially and have not been purified, unless otherwise stated. The melting point (M.p.) of each compound was determined using an MP90 digital melting point apparatus (Mettler Toledo, Columbus, OH, USA) and was not corrected. Thin layer chromatography (TLC) was performed on silica gel 60 F254 aluminum sheets (Merck, Darmstadt, Germany). A Bruker NMR spectrometer (Bruker, Billerica, MA, USA) was used for ^1^H nuclear magnetic resonance (NMR) and ^13^C NMR spectra, while a JEOL JMS-700 Station/JMS-BU-20-GCmate (JEOL, Akishima, Tokyo) was used for high-resolution mass spectra (HRMS).

#### 4.1.1. Synthesis of (E)-3-(2-(4-Chlorophenyl)pyrimidin-5-yl)-1-(3,4-dimethoxyphenyl)prop-2-en-1-one (**B-4**)

Yield: 82%. M.p. 189.6 °C

2-(4-Chlorophenyl)pyrimidine-5-carbaldehyde (1 mmol) was added to 1-(3,4-dimethoxyphenyl)ethan-1-one (1 mmol) and NaOH (1.1 mmol) in ethanol. The final mixture was stirred for 24 h under room temperature (r.t.) conditions. After TLC control, the mixture was transferred to crushed ice. Precipitation was filtered, waterwashed, and dried. The final product was crystalized from ethanol [55].

^1^H NMR (500 MHz, *CDCl*_3_) *δ* (ppm): 3.98 (6H, s, OCH_3_), 6.96 (1H, d, *J* = 10.90 Hz, aromatic), 7.48 (2H, d, *J* = 10.80 Hz, aromatic), 7.64 (1H, s, aromatic), 7.69–7.74 (3H, m, C_2_-H, C_3_-H ve aromatic), 8.44 (2H, d, *J* = 8.35 Hz, aromatic), 9.02 (2H, s, aromatic). ^13^C NMR (125 MHz, *CDCl*_3_) *δ* (ppm): 56.05 (2CH_3_, OCH_3_), 109.99 (CH, aromatic), 110.87 (CH, aromatic), 123.34 (CH, aromatic), 124.16 (CH, C_2_-H), 129.10 (2CH, aromatic), 130.00 (2CH, aromatic), 130.58 (C, aromatic), 135.51 (C, aromatic), 136.50 (C, aromatic), 138.01 (C, aromatic), 144.89 (CH, C_3_-H), 149.65 (C, aromatic), 154.01 (C, aromatic), 156.48 (2CH, aromatic), 164.21 (C, aromatic), 187.48 (C, C=O). HRMS (FAB) calcd. for C_21_H_18_O_3_N_2_Cl [M+H]^+^: m/z = 381.1006; found: 381.0992. (Spectral Data: Supplementary Information. Figures S2–S4).

#### 4.1.2. Synthesis of 5-(2-(4-Chlorophenyl)pyrimidin-5-yl)-3-(3,4-dimethoxyphenyl)-4,5-dihydro-1H-pyrazole-1-carbothioamide (**B-9**)

Yield: 76%. M.p. 200.3 °C

The mixture of **B-4** (3 mmol), thiosemicarbazide (4.5 mmol), and sodium hydroxide (3 mmol) in ethanol was allowed to reflux for 8–12 h. The solution was placed in crushed ice and cooled before washing with water. Precipitation was filtered, waterwashed, and dried. The final product was crystalized from ethanol [55].

^1^H NMR (500 MHz, *CDCl*_3_) *δ* (ppm): 3.23 (1H, dd, *J_AB_* = 17.55 Hz, *J_AX_* = 3.70 Hz, C_4_-H_A_ pyrazoline), 3.83–3.92 (1H, m, C_4_-H_B_ pyrazoline), 3.92 (3H, s, OCH_3_), 3.93 (3H, s, OCH_3_), 6.05 (1H, dd, *J_BX_* = 11.35 Hz, *J_AX_* = 3.70 Hz, C_5_-H_X_ pyrazoline), 6.86 (1H, d, *J* = 9.10 Hz, aromatic), 7.12 (1H, d, *J* = 10.00 Hz, aromatic), 7.35–7.43 (3H, m, aromatic), 8.32 (2H, d, *J* = 7.55 Hz, aromatic), 8.71 (2H, s, NH_2_). ^13^C NMR (125 MHz, *CDCl*_3_) *δ* (ppm): 42.44 (CH_2_, C_4_ pyrazoline), 56.10 (2CH_3_, OCH_3_), 59.23 (CH, C_5_ pyrazoline), 108.54 (CH, aromatic), 110.89 (CH, aromatic), 121.47 (CH, aromatic), 122.72 (C, aromatic), 128.85 (2CH, aromatic), 129.56 (2CH, aromatic), 132.96 (C, aromatic), 135.44 (C, aromatic), 136.87 (C, aromatic), 137.87 (C, aromatic), 149.48 (C, aromatic), 152.35 (C, C_3_ pyrazoline), 155.48 (2CH, aromatic), 162.99 (C, aromatic), 176.05 (C, C=S). HRMS (FAB) calcd. for C_22_H_21_O_2_N_5_ClS [M+H]^+^: m/z = 454.1104; found: 454.1113. (Spectral Data: Supplementary Information. Figures S5–S7).

#### 4.1.3. Synthesis of 2-(5-(2-(4-Chlorophenyl)pyrimidin-5-yl)-3-(3,4-dimethoxyphenyl)-4,5-dihydro-1H-pyrazol-1-yl)-4-(aryl)thiazoles (**BH1-7**)

**B-9** (0.8 mmol) and 2-bromo-1-arylethanone (0.8 mmol) were refluxed in ethanol for 2–6 h. Precipitation was filtered, waterwashed, and dried. The final product was crystalized from ethanol [55].

2-(5-(2-(4-Chlorophenyl)pyrimidin-5-yl)-3-(3,4-dimethoxyphenyl)-4,5-dihydro-1*H*-pyrazol-1-yl)-4-(phenyl)thiazole (**BH-1**)

Yield: 75%. M.p. 217.3 °C

^1^H NMR (500 MHz, *CDCl*_3_) *δ* (ppm): 3.35 (1H, dd, *J_AB_* = 17.15 Hz, *J_AX_* = 7.25 Hz, C_4_-H_A_ pyrazoline), 3.92 (3H, s, OCH_3_), 3.90–3.95 (1H, m, C_4_-H_B_ pyrazoline), 3.98 (3H, s, OCH_3_), 5.65 (1H, dd, *J_BX_* = 11.90 Hz, *J_AX_* = 7.25 Hz, C_5_-H_X_ pyrazoline), 6.85 (1H, s, aromatic), 6.88 (1H, d, *J* = 8.85 Hz, aromatic), 7.12 (1H, d, *J* = 8.25 Hz, aromatic), 7.22 (1H, d, *J* = 7.35 Hz, aromatic), 7.31 (2H, d, *J* = 7.95 Hz, aromatic), 7.42 (3H, d, *J* = 8.88 Hz, aromatic), 7.47 (1H, s, aromatic), 7.65–7.68 (2H, m), 8.36 (2H, d, *J* = 8.70 Hz, aromatic), 8.89 (1H, s, aromatic). ^13^C NMR (125 MHz, *CDCl*_3_) *δ* (ppm): 42.52 (CH_2_, pyrazoline C_4_), 56.00 (CH_3_, OCH_3_), 56.14 (CH_3_, OCH_3_), 60.95 (CH, pyrazoline C_5_), 103.97 (CH, thiazole C_5_), 108.45 (CH, aromatic), 111.11 (CH, aromatic), 120.37 (CH, aromatic), 123.74 (C, aromatic), 125.84 (2CH, aromatic), 127.68 (CH, aromatic), 128.58 (2CH, aromatic), 128.90 (2CH, aromatic), 129.54 (2CH, aromatic), 132.56 (C, aromatic), 134.63 (C, aromatic), 135.90 (C, aromatic), 137.05 (C, aromatic), 149.34 (C, aromatic), 151.17 (C, thiazole C_4_), 151.60 (C, aromatic), 151.84 (C, pyrazoline C_3_), 156.29 (2CH, aromatic), 163.13 (C, aromatic), 165.08 (C, thiazole C_2_). HRMS (FAB) calcd. for C_30_H_25_O_2_N_5_ClS [M+H]^+^: m/z = 554.1417; found: 554.1403. (Spectral Data: Supplementary Information. Figures S8–S10).

2-(5-(2-(4-Chlorophenyl)pyrimidin-5-yl)-3-(3,4-dimethoxyphenyl)-4,5-dihydro-1*H*-pyrazol-1-yl)-4-(4-(nitro)phenyl)thiazole (**BH-2**)

Yield: 71%. M.p. 208.8 °C

^1^H NMR (500 MHz, *CDCl*_3_) *δ* (ppm): 3.42 (1H, dd, *J_AB_* = 17.75 Hz, *J_AX_* = 7.60 Hz, C_4_-H_A_ pyrazoline), 3.95 (3H, s, OCH_3_), 4.00 (3H, s, OCH_3_), 4.02–4.05 (1H, m, C_4_-H_B_ pyrazoline), 5.68 (1H, dd, *J_BX_* = 12.10 Hz, *J_AX_* = 7.10 Hz, C_5_-H_X_ pyrazoline), 6.91 (1H, d, *J* = 8.10 Hz, aromatic), 7.08 (1H, s, aromatic), 7.17 (1H, d, *J* = 10.00 Hz, aromatic), 7.45 (2H, d, *J* = 8.60 Hz, aromatic), 7.49 (1H, s), 7.80 (2H, d, *J* = 8.80 Hz, aromatic), 8.19 (2H, d, *J* = 9.85 Hz, aromatic), 8.39 (2H, d, *J* = 9.85 Hz, aromatic), 8.90 (2H, s, aromatic). ^13^C NMR (125 MHz, *CDCl*_3_) *δ* (ppm): 42.99 (CH_2_, pyrazoline C_4_), 55.78 (2CH_3_, OCH_3_), 60.39 (CH, pyrazoline C_5_), 108.14 (CH, thiazole C_5_), 114.24 (2CH, aromatic), 123.26 (CH, aromatic), 124.18 (C, aromatic), 126.27 (2CH, aromatic), 128.25 (2CH, aromatic), 128.93 (2CH, aromatic), 129.71 (2CH, aromatic), 132.32 (C, aromatic), 135.71 (C, aromatic), 137.27 (C, aromatic), 140.61 (C, aromatic), 146.86 (C, aromatic), 149.57 (C, thiazole C_4_), 152.28 (C, aromatic), 156.40 (2CH, aromatic), 160.31 (C, pyrazoline C_3_), 161.56 (C, aromatic), 163.24 (C, aromatic), 165.42 (C, thiazole C_2_). HRMS (FAB) calcd. for C_30_H_24_O_4_N_6_ClS [M+H]^+^: m/z = 599.1268; found: 599.1232. (Spectral Data: Supplementary Information. Figures S11–S13).

2-(5-(2-(4-Chlorophenyl)pyrimidin-5-yl)-3-(3,4-dimethoxyphenyl)-4,5-dihydro-1*H*-pyrazol-1-yl)-4-(4-(fluoro)phenyl)thiazole (**BH-3**)

Yield: 72%. M.p. 211.6 °C

^1^H NMR (500 MHz, *CDCl*_3_) *δ* (ppm): 3.36 (1H, dd, *J_AB_* = 17.35 Hz, *J_AX_* = 7.10 Hz, C_4_-H_A_ pyrazoline), 3.93 (3H, s, OCH_3_), 3.95–3.97 (1H, m, C_4_-H_B_ pyrazoline), 3.99 (3H, s, OCH_3_), 5.65 (1H, dd, *J_BX_* = 11.95 Hz, *J_AX_* = 7.05 Hz, C_5_-H_X_ pyrazoline), 6.77 (1H, s, aromatic), 6.88 (1H, d, *J* = 7.65 Hz, aromatic), 6.99 (2H, t, *J* = 9.55 Hz, aromatic), 7.13 (1H, d, *J* = 8.55 Hz, aromatic), 7.43 (2H, d, *J* = 8.10 Hz, aromatic), 7.47 (1H, s, aromatic), 7.60–7.63 (2H, m, aromatic), 8.37 (2H, d, *J* = 9.05 Hz, aromatic), 8.88 (2H, s, aromatic). ^13^C NMR (125 MHz, *CDCl*_3_) *δ* (ppm): 42.60 (CH_2_, pyrazoline C_4_), 56.07 (2CH_3_, OCH_3_), 60.49 (CH, pyrazoline C_5_), 103.50 (CH, thiazole C_5_), 108.72 (C, aromatic), 111.53 (2CH, aromatic), 115.55 (C, aromatic), 120.32 (CH, aromatic), 123.73 (CH, aromatic), 127.62 (CH, aromatic), 128.89 (3CH, aromatic), 129.63 (3CH, aromatic), 132.52 (C, aromatic), 135.75 (C, aromatic), 137.24 (C, aromatic), 149.49 (C, aromatic), 150.62 (C, thiazole C_4_), 151.24 (C, aromatic), 151.98 (C, pyrazoline C_3_), 156.18 (2CH, aromatic), 158.10 (C, aromatic), 163.31 (C, aromatic), 165.15 (C, thiazole C_2_). HRMS (FAB) calcd. for C_30_H_23_O_2_N_5_ClFS [M+H]^+^: m/z = 572.1323; found: 571.1243. (Spectral Data: Supplementary Information. Figures S14–S16).

2-(5-(2-(4-Chlorophenyl)pyrimidin-5-yl)-3-(3,4-dimethoxyphenyl)-4,5-dihydro-1*H*-pyrazol-1-yl)-4-(4-(chloro)phenyl)thiazole (**BH-4**)

Yield: 80%. M.p. 187.8 °C

^1^H NMR (500 MHz, *CDCl*_3_) *δ* (ppm): 3.66 (1H, dd, *J_AB_* = 17.65 Hz, *J_AX_* = 7.80 Hz, C_4_-H_A_ pyrazoline), 3.83 (3H, s, OCH_3_), 3.85 (3H, s, OCH_3_), 4.09 (1H, dd, *J_BA_* = 17.65 Hz, *J_BX_ =* 11.70, Hz, C_4_-H_B_ pyrazoline), 5.75 (1H, dd, *J_BX_* = 11.70 Hz, *J_AX_* = 7.85 Hz, C_5_-H_X_ pyrazoline), 7.07 (1H, d, *J* = 8.30 Hz, aromatic), 7.33 (2H, t, *J* = 8.15 Hz, aromatic), 7.40 (3H, d, *J* = 8.70 Hz, aromatic), 7.43 (1H, s, aromatic), 7.58 (2H, d, *J* = 8.85 Hz, aromatic), 7.72 (2H, d, *J* = 8.50 Hz, aromatic), 8.38 (2H, d, *J* = 8.55 Hz, aromatic), 9.03 (2H, s, aromatic). ^13^C NMR (125 MHz, *CDCl*_3_) *δ* (ppm): 42.92 (CH_2_, pyrazoline C_4_), 55.95 (2CH_3_, OCH_3_), 60.70 (CH, pyrazoline C_5_), 104.24 (CH, thiazole C_5_), 108.53 (CH, aromatic), 110.98 (CH, aromatic), 120.18 (C, aromatic), 123.86 (CH, aromatic), 127.12 (2CH, aromatic), 128.71 (2CH, aromatic), 128.84 (2CH, aromatic), 129.59 (2CH, aromatic), 132.38 (C, aromatic), 133.20 (C, aromatic), 133.78 (C, aromatic), 135.72 (C, aromatic), 137.11 (C, aromatic), 149.40 (C, thiazole C_4_), 150.52 (C, pyrazoline C_3_), 151.28 (C, aromatic), 151.92 (C, aromatic), 156.19 (2CH, aromatic), 163.37 (C, aromatic), 165.06 (C, thiazole C_2_). HRMS (FAB) calcd. for C_30_H_24_O_2_N_5_Cl_2_S [M+H]^+^: *m*/*z* = 588.1028; found: 587.0917. (Spectral Data: Supplementary Information. Figures S17–S19).

2-(5-(2-(4-Chlorophenyl)pyrimidin-5-yl)-3-(3,4-dimethoxyphenyl)-4,5-dihydro-1*H*-pyrazol-1-yl)-4-(4-(bromo)phenyl)thiazole (**BH-5**)

Yield: 83%. M.p. 189.7 °C

^1^H NMR (500 MHz, *CDCl*_3_) *δ* (ppm): 3.38 (1H, dd, *J_AB_* = 17.35 Hz, *J_AX_* = 7.15 Hz, C_4_-H_A_ pyrazoline), 3.94 (3H, s, OCH_3_), 3.99 (3H, s, OCH_3_), 3.99–4.04 (1H, m, C_4_-H_B_ pyrazoline), 5.66 (1H, dd, *J_BX_* = 11.90 Hz, *J_AX_* = 7.25 Hz, C_5_-H_X_ pyrazoline), 6.86 (1H, s, aromatic), 6.90 (1H, d, *J* = 8.40 Hz, aromatic), 7.15 (1H, d, *J* = 8.05 Hz, aromatic), 7.43–7.45 (4H, m, aromatic), 7.48 (1H, s, aromatic), 7.53 (2H, d, *J* = 8.75 Hz, aromatic), 8.38 (2H, d, *J* = 8.65 Hz, aromatic), 8.89 (2H, s, aromatic). ^13^C NMR (125 MHz, *CDCl*_3_) *δ* (ppm): 42.68 (CH_2_, pyrazoline C_4_), 56.04 (2CH_3_, OCH_3_), 60.95 (CH, pyrazoline C_5_), 104.36 (CH, thiazole C_5_), 108.65 (CH, aromatic), 110.98 (CH, aromatic), 120.30 (CH, aromatic), 121.53 (C, aromatic), 123.61 (C, aromatic), 127.29 (2CH, aromatic), 128.89 (2CH, aromatic), 129.74 (2CH, aromatic), 131.95 (2CH, aromatic), 132.33 (C, aromatic), 133.55 (C, aromatic), 135.63 (C, aromatic), 137.35 (C, aromatic), 149.40 (C, aromatic), 150.49 (C, thiazole C_4_), 151.25 (C, pyrazoline C_3_), 152.05 (C, aromatic), 156.21 (2CH, aromatic), 157.58 (C, aromatic), 165.20 (C, thiazole C_2_). HRMS (FAB) calcd. for C_30_H_24_O_2_N_5_ClBrS [M+H]^+^: m/z = 632.0523; found: 632.0474. (Spectral Data: Supplementary Information. Figures S20–S22).

2-(5-(2-(4-Chlorophenyl)pyrimidin-5-yl)-3-(3,4-dimethoxyphenyl)-4,5-dihydro-1*H*-pyrazol-1-yl)-4-(4-(cyano)phenyl)thiazole (**BH-6**)

Yield: 82%. M.p. 205.5 °C

^1^H NMR (500 MHz, *CDCl*_3_) *δ* (ppm): 3.40 (1H, dd, *J_AB_* = 17.40 Hz, *J_AX_* = 7.25 Hz, C_4_-H_A_ pyrazoline), 3.95 (3H, s, OCH_3_), 3.99 (3H, s, OCH_3_), 4.01–4.04 (1H, m, C_4_-H_B_ pyrazoline), 5.67 (1H, dd, *J_BX_* = 12.40 Hz, *J_AX_* = 7.55 Hz, C_5_-H_X_ pyrazoline), 6.91 (1H, d, *J* = 8.10 Hz, aromatic), 7.01 (1H, s, aromatic), 7.16 (1H, d, *J* = 8.40 Hz, aromatic), 7.45 (2H, d, *J* = 9.05 Hz, aromatic), 7.48 (1H, s, aromatic), 7.60 (2H, d, *J* = 8.60 Hz, aromatic), 7.74 (2H, d, *J* = 8.60 Hz, aromatic), 8.38 (2H, d, *J* = 8.60 Hz, aromatic), 8.89 (2H, s, aromatic). ^13^C NMR (125 MHz, *CDCl*_3_) *δ* (ppm): 42.77 (CH_2_, pyrazoline C_4_), 55.95 (2CH_3_, OCH_3_), 60.34 (CH, pyrazoline C_5_), 106.99 (CH, thiazole C_5_), 109.06 (CH, aromatic), 110.87 (CH, aromatic), 120.43 (CH, aromatic), 122.11 (C, aromatic), 123.79 (C, aromatic), 126.37 (2C, aromatic), 128.96 (3CH, aromatic), 129.61 (3CH, aromatic), 132.84 (2CH, aromatic), 135.29 (C, aromatic), 137.09 (C, aromatic), 139.04 (C, aromatic), 149.46 (C, aromatic), 149.75 (C, thiazole C_4_), 150.36 (C, aromatic), 152.48 (C, pyrazoline C_3_), 156.48 (2CH, aromatic), 161.52 (C, aromatic), 165.40 (C, thiazole C_2_). HRMS (FAB) calcd. for C_31_H_23_O_2_N_6_ClS [M+H]^+^: m/z = 578.1292; found: 578.1288. (Spectral Data: Supplementary Information. Figures S23–S25).

2-(5-(2-(4-Chlorophenyl)pyrimidin-5-yl)-3-(3,4-dimethoxyphenyl)-4,5-dihydro-1*H*-pyrazol-1-yl)-4-(4-(trifluoromethyl)phenyl)thiazole (**BH-7**)

Yield: 78%. M.p. 187.2 °C

^1^H NMR (500 MHz, *CDCl*_3_) *δ* (ppm): 3.39 (1H, dd, *J_AB_* = 17.20 Hz, *J_AX_* = 6.55 Hz, C_4_-H_A_ pyrazoline), 3.85–3.92 (1H, m, C_4_-H_B_ pyrazoline), 3.94 (3H, s, OCH_3_), 3.99 (3H, s, OCH_3_), 5.67 (1H, dd, *J_BX_* = 12.00 Hz, *J_AX_* = 7.20 Hz, C_5_-H_X_ pyrazoline), 6.97 (1H, s, aromatic), 7.16 (1H, d, *J* = 8.55 Hz, aromatic), 7.43–7.49 (4H, m, aromatic), 7.58 (2H, t, *J* = 8.25 Hz, aromatic), 7.76 (2H, t, *J* = 10.50 Hz, aromatic), 8.37–8.42 (2H, m, aromatic), 8.89 (2H, s, aromatic). ^13^C NMR (125 MHz, *CDCl*_3_) *δ* (ppm): 42.42 (CH_2_, pyrazoline C_4_), 58.79 (2CH_3_, OCH_3_), 60.25 (CH, pyrazoline C_5_), 106.97 (CH, thiazole C_5_), 108.66 (CH, aromatic), 110.66 (CH, aromatic), 120.34 (CH, aromatic), 122.61 (C, aromatic), 123.42 (C, aromatic), 125.87 (2C, aromatic), 128.13 (2CH, aromatic), 128.79 (2CH, aromatic), 129.87 (2CH, aromatic), 132.79 (2CH, aromatic), 134.48 (C, aromatic), 135.70 (C, aromatic), 138.94 (C, aromatic), 148.92 (C, aromatic), 146.41 (C, thiazole C_4_), 151.37 (C, pyrazoline C_3_), 152.30 (C, aromatic), 155.99 (2CH, aromatic), 160.36 (C, aromatic), 164.09 (C, thiazole C_2_). HRMS (FAB) calcd. for C_31_H_23_O_2_N_5_ClF_3_S [M+H]^+^: m/z = 621.1213; found: 621.1205. (Spectral Data: Supplementary Information. Figures S26–S28).

### 4.2. Cell Culture and Drug Treatment

Dulbecco’s modified Eagle’s medium (DMEM) (Wako Pure Chemical Industries, Osaka, Japan) containing 10% fetal bovine serum (FBS) (Equitech-Bio, Kerrville, TX, USA) was used for the culture of A549 and MCF-7 human cancer cells, while RPMI 1640 (Wako Pure Chemical Industries) containing 10% FBS was used for the culture of Jurkat cells, derived from human leukemia. On the other hand, PBMCs (Precision Bioservices, Frederick, MD, USA) were incubated in RPMI 1640 supplemented with 10% human serum AB (HS) (Gemini, Woodland, CA, USA). All media also incorporated 89 μg/mL of streptomycin (Meiji Seika Pharma, Tokyo, Japan), and cells were maintained at 37 °C in a 95% air and 5% CO_2_ humidified chamber. The microtiter tissue culture plates (24-well and 96-well) (Iwaki, Asahi Glass Co., Chiba, Japan) were used for cancer cells (4 × 10^4^ cells/mL) and PBMCs (5 × 10^5^ cells/mL), respectively. Prior to drug treatment, all of these cells were incubated for 72 h. The optimal cell density for cytotoxicity testing was established in preliminary experiments. The stock solutions of compounds and lapatinib (Sigma-Aldrich, St. Louis, MO, USA) (0.1 mM, 0.3 mM, 1 mM, 3 mM, and 10 mM) were prepared in dimethyl sulfoxide (DMSO; Wako Pure Chemical Industries), then applied to fresh culture medium. The final concentration of DMSO in the culture medium was adjusted to 1% [61,62,63].

### 4.3. MTT Assay for Cytotoxicity of Compounds

The cellular reduction in MTT (Dojindo Molecular Technologies, Kumamoto, Japan) was measured as described in the literature with minor modifications [61,62,63]. The compounds and lapatinib were incubated with cells at final concentrations ranging from 1 to 100 μM for 72 h. MTT was then added at a final concentration 0.275 mg/mL and incubated for a further 4 h at 37 °C. The medium was removed and 100 μL DMSO was added to each well to dissolve the formazan crystals. The dissolved crystals were diluted (1:10) with DMSO, and 100 μL of the solution was transferred to a 96-well microtiter plate (Iwaki, Asahi Glass Co.). Absorbance was measured at 570 nm using an Infinitive M1000 microplate spectrophotometer (Tecan, Groding, Austria). Each concentration was tested in triplicate, and the IC_50_ values were determined as the drug concentrations that reduced absorbance to 50% of the control values.

### 4.4. Detection of Apoptotic and Necrotic Cells

Compound **B-4** at an IC_50_ concentration was administered to A549 and MCF-7 cells for 24 h. After detachment from the plate with 0.05% trypsin and washing twice with 1× binding buffer, the cells were incubated for 20 min at r.t. in the dark with a staining solution bearing 50 μL 1× binding buffer, 4 μL FTIC annexin V solution, 4 μL ethidium homodimer III solution, and 4 μL Hoechst 33342 solution. The cells were washed with 1× binding buffer, fixed with 2% paraformaldehyde, washed with PBS, and analyzed by a Biorevo Fluorescence BZ-9000 (Keyence, Osaka, Japan) integrated fluorescence microscope [61,62,63].

### 4.5. EGFR Inhibition Assay

The kinase profiling assay protocol (EGFR Kinase Enzyme System, V3831) was performed following the manufacturer’s instructions (Promega Corporation, Madison, WI, USA) with minor modifications [55]. As per the protocol, 95 μL of 2.5× kinase buffer and 15 μL of 100 μM ATP solution were used for the dilution of the EGFR kinase (human, recombinant; amino acids 695–end) and its substrate (4:1 Glu, Tyr). Kinase reactions were performed in a 384-well plate in the presence of 2 μL of kinase working stock, 2 μL of ATP/substrate working stock, and 1 μL of the test compound solution (1 μM and 1 μM) or 5% DMSO solution. After incubating for 2 h at r.t., kinase activity was assessed using the ADP-Glo Kinase Assay (Promega Corporation, Madison, WI, USA) following the manufacturer’s instructions. Specifically, 5 μL of ADP-Glo Reagent was added to each well and incubated for 40 min, followed by the addition of 10 μL of Kinase Detection Reagent and further incubation for 30 min at room temperature. Luminescence was measured using the Infinite M1000 microplate reader (Tecan, Grödig, Austria) with an integration time of 0.5–1 s to evaluate the kinase inhibitory effects of the compounds in a dose-dependent manner.

### 4.6. Molecular Docking

A high resolution (1.50 Å) X-ray structure of the EGFR complexed with and inhibitor (tak-285) was downloaded from the Protein Data Bank (PDB ID: 3POZ) [64,65] and was prepared for molecular docking simulation using Schrödinger’s 2022-4 drug discovery software. While preparing the protein, the OPSL4 force filed was applied, with a convergence RMSD cut-off of 0.30 Å for energy minimization, to ensure the stability and quality of the resultant protein for subsequent studies.

A grid box was generated centered on the native ligand tak-285 (03P) to identify the binding site of the EGFR. All the docking simulations were carried out using induced fit docking (IFD) protocol [66,67,68] with default settings.

### 4.7. MD Simulations

Flare™ v9.0.0 [69,70,71,72] software by Cresset was used for molecular dynamics simulations, which is based on the OpenMM package [73]. An orthogonal solvent box was generated using three point extended simple point charge (SPC/E) [74] explicit water solvent model. AMBER was employed as the force field used during the calculation, and the GAFF2 version was selected for small molecules. Seven sodium ions were placed as counterions to neutralize the system. The ionic strength of the explicit solvent box was adjusted to 0.15 M NaCl. The Particle Mesh Ewald (PME) method was used to consider all long-range electrostatic interactions. The reporting frequency (number of time steps between trajectory frames) was adjusted to 20,000. Periodic boundary conditions were applied and finally, the production stage was run for 300 ns under an isothermal–isobaric ensemble (NPT).

### 4.8. In Silico ADME Prediction

QikProp [59] and the SwissADME web tool [60] were used to estimate the pharmacokinetic determinants of **B-4**, as previously explained [75,76,77].

## 5. Conclusions

New pyrimidine-containing compounds (chalcone derivative (**B-4**), pyrazoline–carbothioamide (**B-9**), and pyrazoline–thiazole hybrids (**BH1-7**)) have been prepared and structurally resolved using our well-established methods. These molecules have been tested in A549 NSCLC and MCF-7 breast cancer cells. Indicating the important role of the chalcone moiety, **B-4** induced cytotoxicity as the most potent anticancer agent against both A549 and MCF-7 cells. In addition, **B-4** revealed a low level of cytotoxicity to healthy cells (PBMCs). **B-4** was shown to induce apoptosis in NSCLC and breast cancer cells and to significantly inhibit the EGFR. Molecular dynamics simulations run for 300 ns revealed strong, favorable, and stable interactions between **B-4** and the EGFR. The potential of **B-4** as a drug candidate for both anti-NSCLC and anti-breast cancer activity studies was highlighted by in silico ADME prediction. **B-4** can be identified as a small-molecule EGFR inhibitor to be effective against NSCLC and breast cancer for future studies.

## Figures and Tables

**Table 1:** The IC_50_ values of **B-4** against A549, MCF-7, Jurkat cells, and PBMCs in comparison to lapatinib.

Compound	IC_50_ Values (μM)				SI *
	A549 Cells	MCF-7 Cells	Jurkat Cells	PBMCs	
**B** **-** **4**	20.49 ± 2.71	6.70 ± 1.02	1.50 ± 0.40	68.89 ± 10.28	45.93
Lapatinib	18.21 ± 3.25	9.71 ± 1.12	1.43 ± 0.35	12.71 ± 2.08	8.89

* Selectivity Index (SI) = IC_50_ for PBMC/IC_50_ for Jurkat cell line.

## Data Availability

The original contributions presented in this study are included in the article/supplementary material. Further inquiries can be directed to the corresponding author.

## Supplementary Materials

The following supporting information can be downloaded at: https://www.mdpi.com/article/10.3390/ph18020270/s1, Figure S1: The superimposed conformations of X-ray co-crystal structure (orange) and the docked pose (green) generated after IFD protocol; Figure S2: ^1^H NMR Spectrum of **B-4**; Figure S3: ^13^C NMR Spectrum of **B-4**; Figure S4: Mass Spectrum of **B-4**; Figure S5: ^1^H NMR Spectrum of **B-9**; Figure S6: ^13^C NMR Spectrum of **B-9**; Figure S7: Mass Spectrum of **B-9**; Figure S8: ^1^H NMR Spectrum of **BH-1**; Figure S9: ^13^C NMR Spectrum of **BH-1**; Figure S10: Mass Spectrum of **BH-1**; Figure S11: ^1^H NMR Spectrum of **BH-2**; Figure S12: ^13^C NMR Spectrum of **BH-2**; Figure S13: Mass Spectrum of **BH-2**; Figure S14: ^1^H NMR Spectrum of **BH-3**; Figure S15: ^13^C NMR Spectrum of **BH-3**; Figure S16: Mass Spectrum of **BH-3**; Figure S17: ^1^H NMR Spectrum of **BH-4**; Figure S18: ^13^C NMR Spectrum of **BH-4**; Figure S19: Mass Spectrum of **BH-4**; Figure S20: ^1^H NMR Spectrum of **BH-5**; Figure S21: ^13^C NMR Spectrum of **BH-5**; Figure S22: Mass Spectrum of **BH-5**; Figure S23: ^1^H NMR Spectrum of **BH-6**; Figure S24: ^13^C NMR Spectrum of **BH-6**; Figure S25: Mass Spectrum of **BH-6**; Figure S26: ^1^H NMR Spectrum of **BH-7**; Figure S27: ^13^C NMR Spectrum of **BH-7**; Figure S28: Mass Spectrum of **BH-7.**
