# Supplementary material for: Design, Synthesis, and Mechanistic Anticancer Evaluation of New Pyrimidine-Tethered Compounds
Source: Pharmaceuticals (Basel). 2025 Feb 19;18(2):270. doi: 10.3390/ph18020270 (PMC11859636; doi:10.3390/ph18020270)
Supplement: Supplementary file 1 [file pharmaceuticals-18-00270-s001.zip › pharmaceuticals-3431308-supplementary.pdf]

Supplementary Information

Design, Synthesis, and Mechanistic Anticancer Evaluation of New Pyrimidine-Tethered Compounds

Farida Reymova [1], Belgin Sever [2,3], Edanur Topalan [4], Canan Sevimli-Gur [5], Mustafa Can [1], Amaç Fatih Tuyun [6], Faika Başoğlu [7], Abdulilah Ece [8], Masami Otsuka [3,9], Mikako Fujita [3], Hasan Demirci [4] and Halilibrahim Ciftci [1,3,9,10,*]

[1] Department of Bioengineering Sciences, Izmir Katip Celebi University, Izmir 35620, Türkiye; feridereymova@gmail.com (F.R.); mustafa.can@ikc.edu.tr (M.C.)
[2] Department of Pharmaceutical Chemistry, Faculty of Pharmacy, Anadolu University, Eskisehir 26470, Türkiye; belginsever@anadolu.edu.tr (B.S.)
[3] Medicinal and Biological Chemistry Science Farm Joint Research Laboratory, Faculty of Life Sciences, Kumamoto University, Kumamoto 862-0973, Japan; motsuka@gpo.kumamoto-u.ac.jp (M.O.); mfujita@kumamoto-u.ac.jp (M.F.)
[4] Department of Molecular Biology and Genetics, Koc University, Istanbul 34450, Türkiye; etopalan23@ku.edu.tr (E.T.); hdemirci@ku.edu.tr (H.D.)
[5] Department of Basic Pharmaceutical Sciences, Faculty of Pharmacy, Izmir Katip Celebi University, Izmir 35620, Türkiye; canansevimli@yahoo.com (C.S.G.)
[6] Department of Chemistry, Faculty of Science, Istanbul University, Fatih, Istanbul 34126, Türkiye; aftuyun@istanbul.edu.tr (A.F.T.)
[7] Department of Pharmaceutical Chemistry, Faculty of Pharmacy, European University of Lefke, Northern Cyprus, TR-10 Mersin 99800, Türkiye; fabasoglu@eul.edu.tr (F.B.)
[8] Department of Medical Biochemistry, Faculty of Medicine, Biruni University, İstanbul 34015, Türkiye; aece@biruni.edu.tr (A.E.)
[9] Department of Drug Discovery, Science Farm Ltd., Kumamoto 862-0976, Japan
[10] Department of Molecular Biology and Genetics, Burdur Mehmet Akif Ersoy University, Istiklal Campus, Burdur 15030, Türkiye

* Correspondence: hciftci@mehmetakif.edu.tr (H.C.)

Supplementary Figures

Figure S1: The superimposed conformations of X-ray co-crystal structure (orange) and the docked pose (green) generated after IFD protocol
Figure S2: ^{1}H NMR Spectrum of **B-4**
Figure S3: ^{13}C NMR Spectrum of **B-4**
Figure S4: Mass Spectrum of **B-4**
Figure S5: ^{1}H NMR Spectrum of **B-9**
Figure S6: ^{13}C NMR Spectrum of **B-9**
Figure S7: Mass Spectrum of **B-9**
Figure S8: ^{1}H NMR Spectrum of **BH-1**
Figure S9: ^{13}C NMR Spectrum of **BH-1**
Figure S10: Mass Spectrum of **BH-1**
Figure S11: ^{1}H NMR Spectrum of **BH-2**
Figure S12: ^{13}C NMR Spectrum of **BH-2**
Figure S13: Mass Spectrum of **BH-2**
Figure S14: ^{1}H NMR Spectrum of **BH-3**
Figure S15: ^{13}C NMR Spectrum of **BH-3**
Figure S16: Mass Spectrum of **BH-3**
Figure S17: ^{1}H NMR Spectrum of **BH-4**
Figure S18: ^{13}C NMR Spectrum of **BH-4**
Figure S19: Mass Spectrum of **BH-4**
Figure S20: ^{1}H NMR Spectrum of **BH-5**
Figure S21: ^{13}C NMR Spectrum of **BH-5**
Figure S22: Mass Spectrum of **BH-5**
Figure S23: ^{1}H NMR Spectrum of **BH-6**
Figure S24: ^{13}C NMR Spectrum of **BH-6**
Figure S25: Mass Spectrum of **BH-6**
Figure S26: ^{1}H NMR Spectrum of **BH-7**
Figure S27: ^{13}C NMR Spectrum of **B BH-7**
Figure S28: Mass Spectrum of **BH-7**

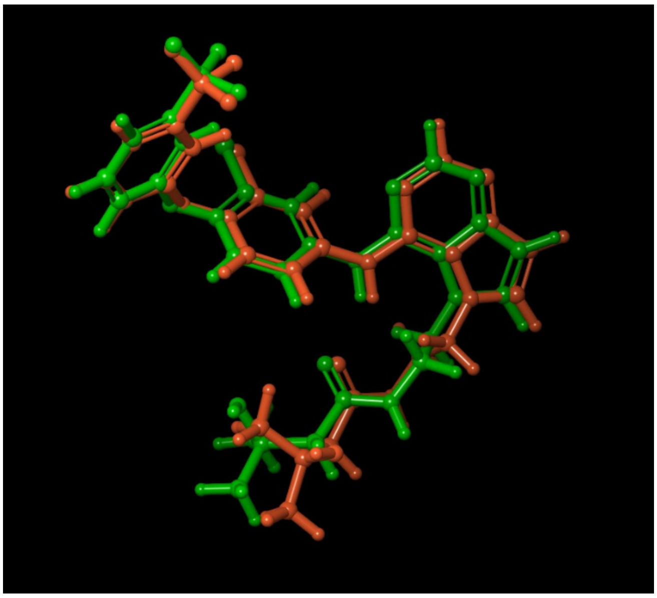

Figure S1. The superimposed conformations of X-ray co-crystal structure (orange) and the docked pose (green) generated after IFD protocol

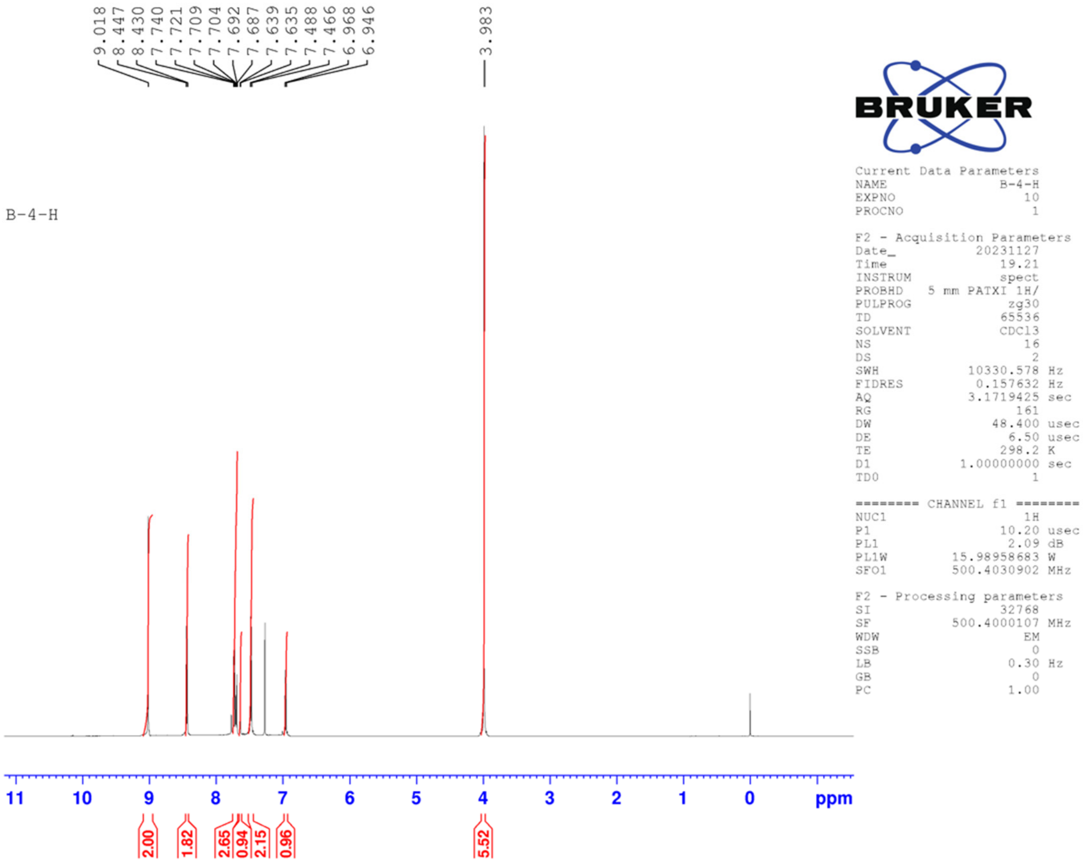

Figure S2. ^{1}H NMR Spectrum of **B-4**

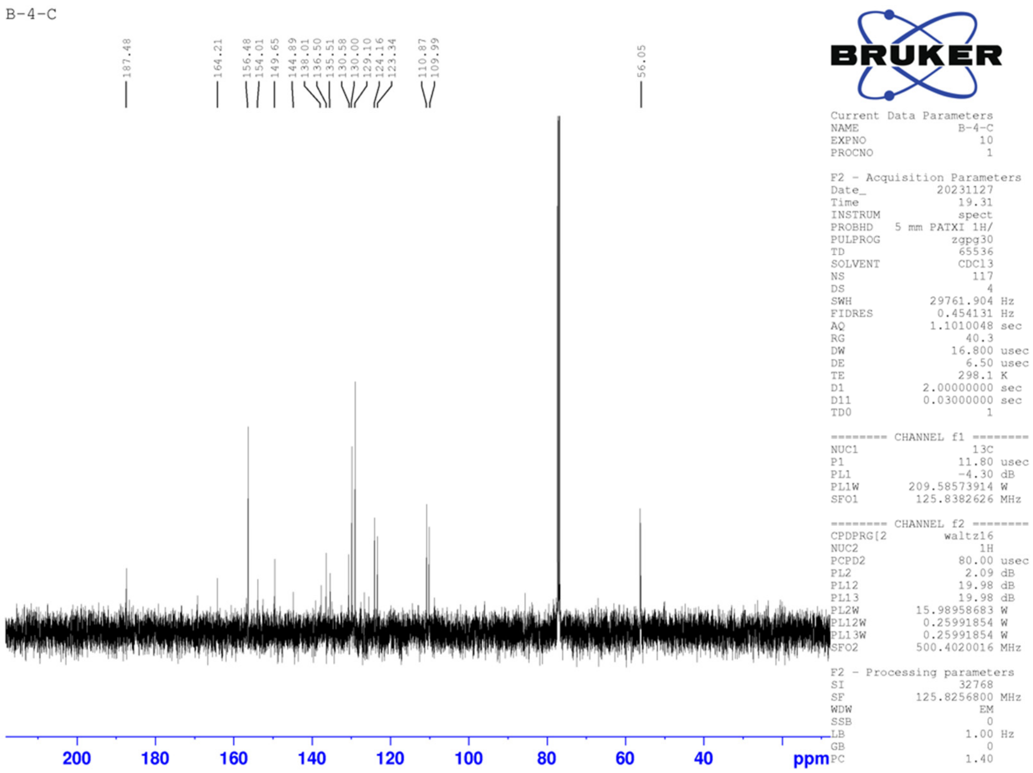

Figure S3. ^{13}C NMR Spectrum of **B-4**

```
[ Elemental Composition ]                                          Page: 1
Data   : gousei820                     Date : 28-Nov-2023 15:47
Sample: B-4/sever belgin
Note : NBA
Inlet : Direct                         Ion Mode : FAB+
RT : 2.00 min                          Scan#: (7,11)
Elements : C 100/0, H 100/0, O 4/2, N 3/1, Cl 2/0
Mass Tolerance      : 20ppm, 10mmu if m/z < 500, 20mmu if m/z > 1000
Unsaturation (U.S.) : -0.5 - 100.0

Observed m/z Int%   Err[ppm / mmu]    U.S.  Composition
  381.0992   63.4      -2.5 /  -1.0   18.0  C 24 H 15 O 4 N
                       -3.8 /  -1.4   13.5  C 21 H 18 O 3 N 2 Cl
                      +24.4 /  +9.3    9.0  C 19 H 21 O 3 N Cl 2
                       -5.1 /  -1.9    9.0  C 18 H 21 O 2 N 3 Cl 2

  380.0917   36.7      -1.4 /  -0.5   18.5  C 24 H 14 O 4 N
                       -2.7 /  -1.0   14.0  C 21 H 17 O 3 N 2 Cl
                      +25.6 /  +9.7    9.5  C 19 H 20 O 3 N Cl 2
                       -4.0 /  -1.5    9.5  C 18 H 20 O 2 N 3 Cl 2
```

```
[ Theoretical Ion Distribution ]                                    Page: 1
Molecular Formula : C21 H18 O3 N2 Cl
                      (m/z   381.1006, MW   381.8385, U.S. 13.5)
Base Peak :    381.1006, Averaged MW :   381.8394(a),    381.8421(w)

    m/z       INT.
  381.1006 100.0000 **********************************************************
  382.1038  24.4759 **************
  383.0985  35.4439 *********************
  384.1012   8.1872 *****
  385.1039   1.1379 *
  386.1065   0.1172
  387.1091   0.0096
  388.1117   0.0007
```

Figure S4: Mass Spectrum of **B-4**

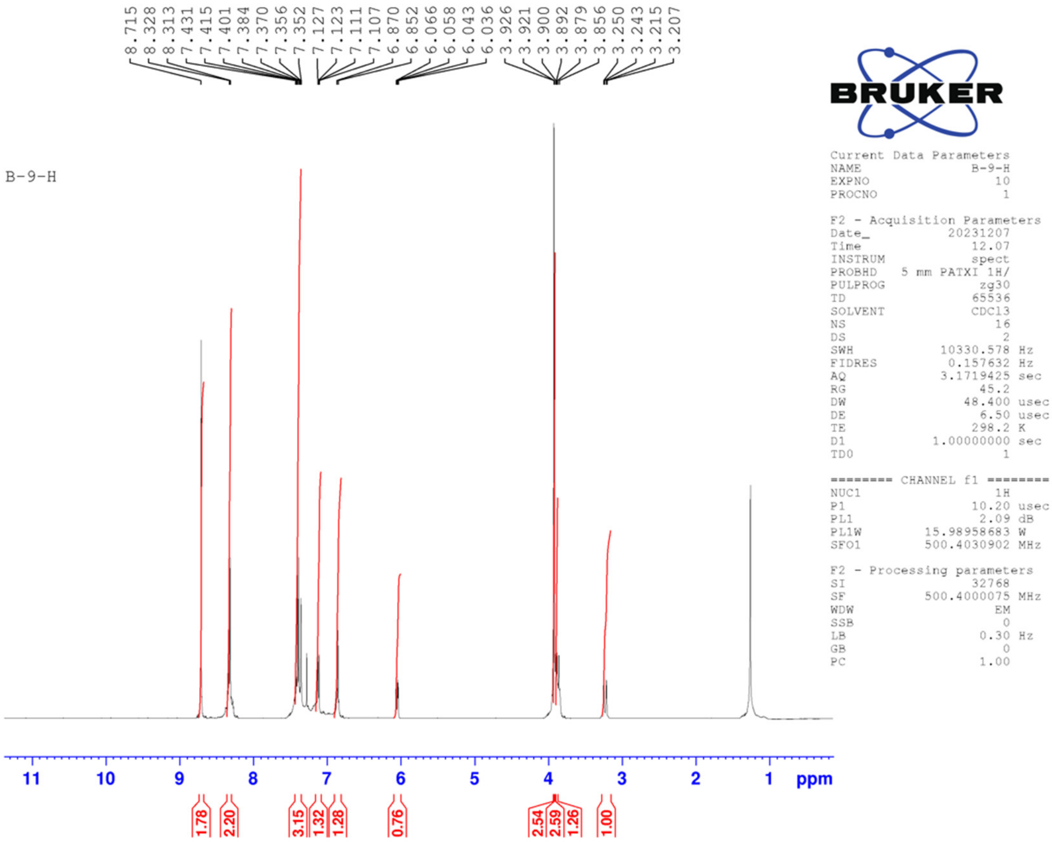

Figure S5: ^{1}H NMR Spectrum of **B-9**

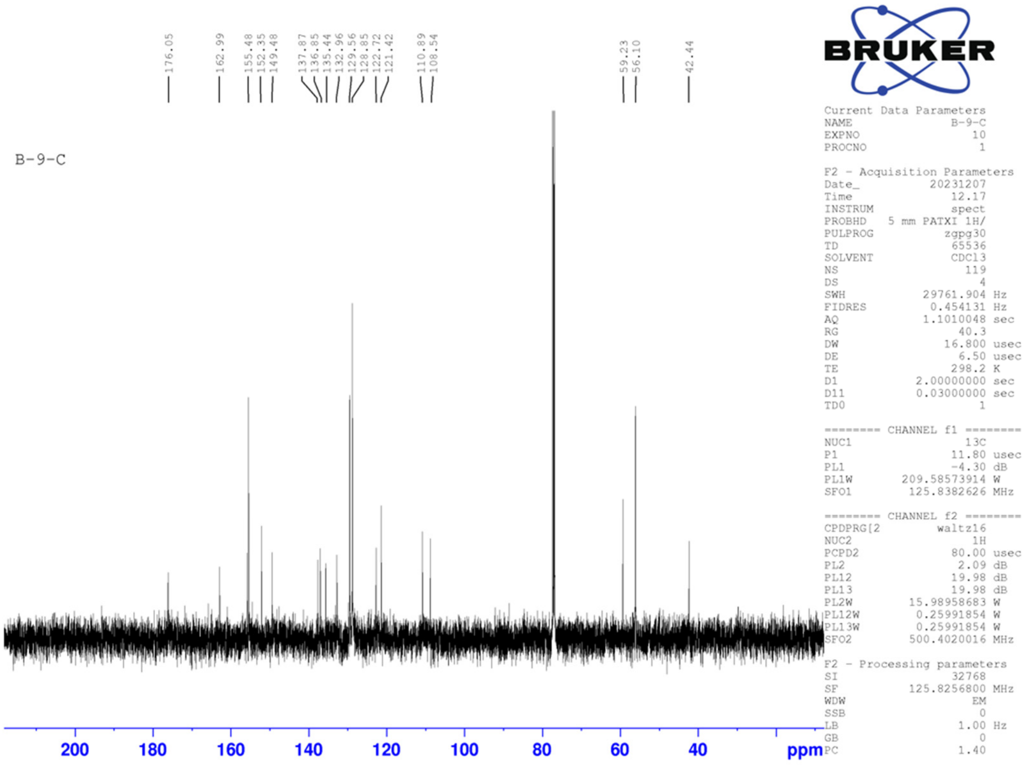

Figure S6: ^{13}C NMR Spectrum of **B-9**

[Elemental Composition] Page: 1
Data : gousei833 Date : 08-Dec-2023 11:48
Sample: B-9/sever belgin
Note : NBA
Inlet : Direct Ion Mode : FAB+
RT : 1.50 min Scan#: (4,10)
Elements : C 100/0, H 100/0, O 3/1, N 6/4, Cl 2/0, S 2/0
Mass Tolerance : 20ppm, 10mmu if m/z < 500, 20mmu if m/z > 1000
Unsaturation (U.S.) : -0.5 - 100.0

Observed m/z	Int%	Err[ppm / mmu]	U.S.	Composition
454.1113	100.0	+10.4 / +4.7	24.0	C 28 H 14 O 3 N 4
		-14.3 / -6.5	24.0	C 27 H 14 O 2 N 6
		-18.3 / -8.3	19.0	C 26 H 19 O 2 N 4 Cl
		+9.4 / +4.3	19.5	C 25 H 17 O 2 N 5 Cl
		-19.4 / -8.8	14.5	C 23 H 22 O N 5 Cl 2
		+8.3 / +3.8	15.0	C 22 H 20 O N 6 Cl 2
		+3.0 / +1.4	20.0	C 25 H 18 O 3 N 4 S
		-21.7 / -9.9	20.0	C 24 H 18 O 2 N 6 S
		+1.9 / +0.9	15.5	C 22 H 21 O 2 N 5 Cl S
		+0.9 / +0.4	11.0	C 19 H 24 O N 6 Cl 2 S
		-4.4 / -2.0	16.0	C 22 H 22 O 3 N 4 S 2
		-5.5 / -2.5	11.5	C 19 H 25 O 2 N 5 Cl S 2
		+18.2 / +8.3	7.0	C 17 H 28 O 2 N 4 Cl 2 S 2
		-6.6 / -3.0	7.0	C 16 H 28 O N 6 Cl 2 S 2

```
[ Theoretical Ion Distribution ]                                    Page: 1
Molecular Formula : C22 H21 O2 N5 Cl S
                       (m/z   454.1104, MW   454.9600, U.S. 15.5)
Base Peak :    454.1104, Averaged MW :   454.9597(a),    454.9623(w)

    m/z        INT.
  454.1104 100.0000 ************************************************************
  455.1133  27.4864 ****************
  456.1082  40.4449 ***********************
  457.1106  10.3906 ******
  458.1083   2.9317 **
  459.1089   0.5369
  460.1100   0.0740
  461.1112   0.0082
  462.1127   0.0007
```

Figure S7: Mass Spectrum of **B-9**

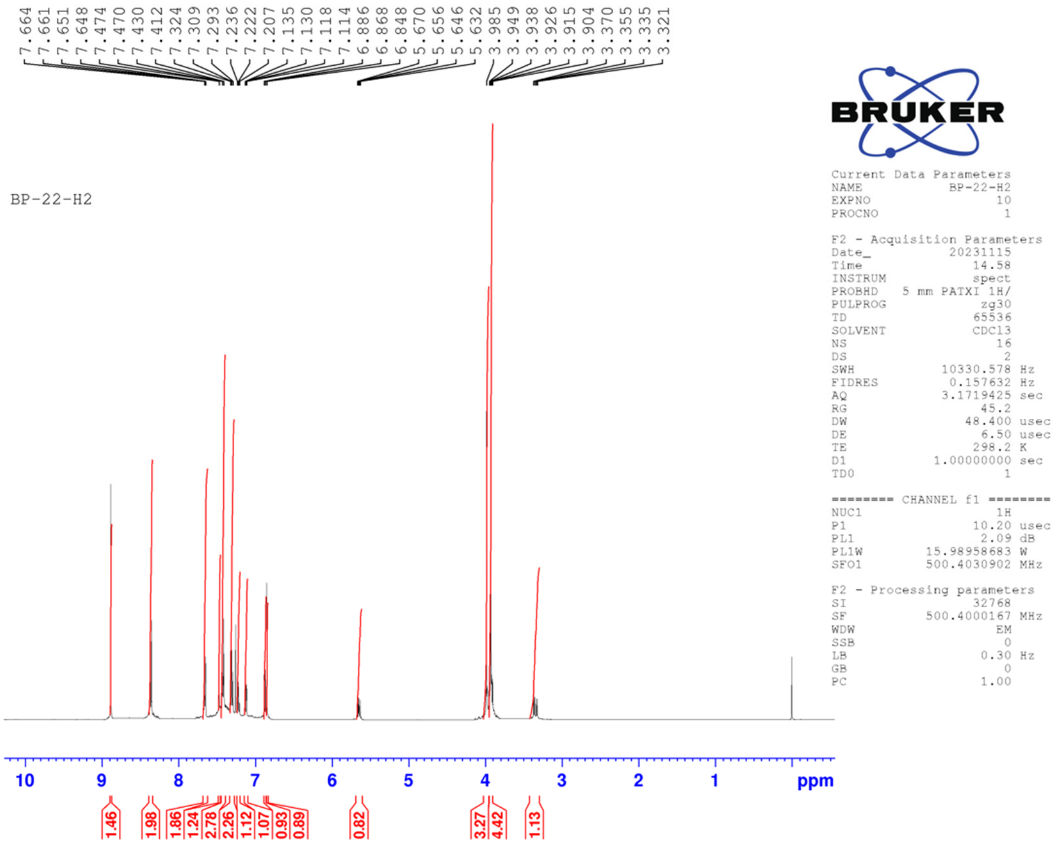

Figure S8: ^{1}H NMR Spectrum of **BH-1**

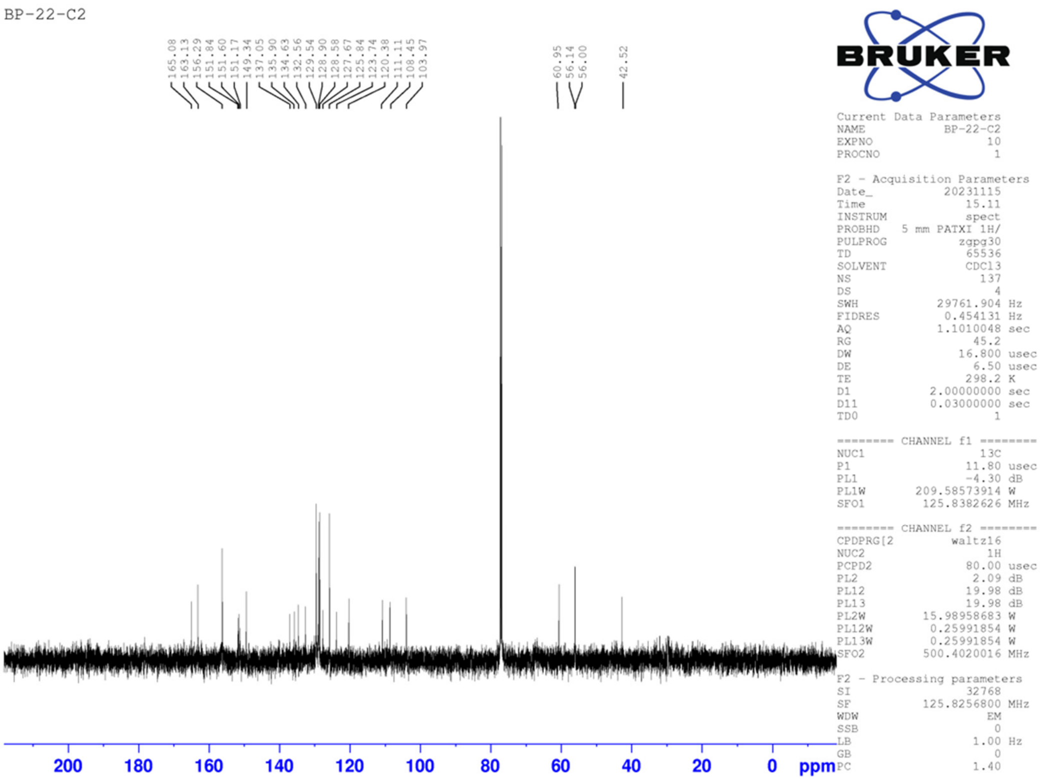

Figure S9: ^{13}C NMR Spectrum of **BH-1**

[Elemental Composition] Page: 1

Data : gousei790 Date : 16-Nov-2023 10:51
Sample: BP-22/sever belgin
Note : NBA
Inlet : Direct Ion Mode : FAB+
RT : 1.13 min Scan#: (4,7)
Elements : C 100/0, H 100/0, O 3/1, N 6/4, Cl 2/0, S 2/0
Mass Tolerance : 10ppm, 10mmu if m/z < 1000, 20mmu if m/z > 2000
Unsaturation (U.S.) : -0.5 - 100.0

Observed m/z	Int%	Err[ppm / mmu]	U.S.	Composition
554.1403	100.0	+4.4 / +2.4	30.0	C 36 H 18 O 3 N 4
		-15.9 / -8.8	30.0	C 35 H 18 O 2 N 6
		+3.5 / +1.9	25.5	C 33 H 21 O 2 N 5 Cl
		+2.6 / +1.5	21.0	C 30 H 24 O N 6 Cl 2
		-1.7 / -0.9	26.0	C 33 H 22 O 3 N 4 S
		-2.6 / -1.4	21.5	C 30 H 25 O 2 N 5 Cl S
		+16.8 / +9.3	17.0	C 28 H 28 O 2 N 4 Cl 2 S
		-3.5 / -1.9	17.0	C 27 H 28 O N 6 Cl 2 S
		-7.8 / -4.3	22.0	C 30 H 26 O 3 N 4 S 2
		+14.9 / +8.3	22.5	C 29 H 24 O 3 N 5 S 2
		-8.7 / -4.8	17.5	C 27 H 29 O 2 N 5 Cl S 2
		+14.0 / +7.8	18.0	C 26 H 27 O 2 N 6 Cl S 2
		+10.7 / +5.9	13.0	C 25 H 32 O 2 N 4 Cl 2 S 2
		-9.5 / -5.3	13.0	C 24 H 32 O N 6 Cl 2 S 2
553.1334	93.2	+6.1 / +3.4	30.5	C 36 H 17 O 3 N 4
		-14.2 / -7.9	30.5	C 35 H 17 O 2 N 6
		-17.5 / -9.7	25.5	C 34 H 22 O 2 N 4 Cl
		+5.2 / +2.9	26.0	C 33 H 20 O 2 N 5 Cl
		+4.3 / +2.4	21.5	C 30 H 23 O N 6 Cl 2
		+0.0 / +0.0	26.5	C 33 H 21 O 3 N 4 S
		-0.9 / -0.5	22.0	C 30 H 24 O 2 N 5 Cl S
		-1.8 / -1.0	17.5	C 27 H 27 O N 6 Cl 2 S
		-6.1 / -3.4	22.5	C 30 H 25 O 3 N 4 S 2
		+16.6 / +9.2	23.0	C 29 H 23 O 3 N 5 S 2
		-7.0 / -3.9	18.0	C 27 H 28 O 2 N 5 Cl S 2
		+15.7 / +8.7	18.5	C 26 H 26 O 2 N 6 Cl S 2
		+12.4 / +6.9	13.5	C 25 H 31 O 2 N 4 Cl 2 S 2
		-7.9 / -4.4	13.5	C 24 H 31 O N 6 Cl 2 S 2

[Theoretical Ion Distribution] Page: 1

Molecular Formula : C30 H25 O2 N5 Cl S
(m/z 554.1417, MW 555.0798, U.S. 21.5)
Base Peak : 554.1417, Averaged MW : 555.0799(a), 555.0821(w)

m/z	INT.	
554.1417	100.0000	**
555.1447	36.4443	**********************
556.1401	43.2588	**************************
557.1422	14.1181	********
558.1412	4.0070	**
559.1415	0.8393	
560.1426	0.1333	
561.1439	0.0169	
562.1455	0.0018	
563.1473	0.0002	

Figure S10: Mass Spectrum of **BH-1**

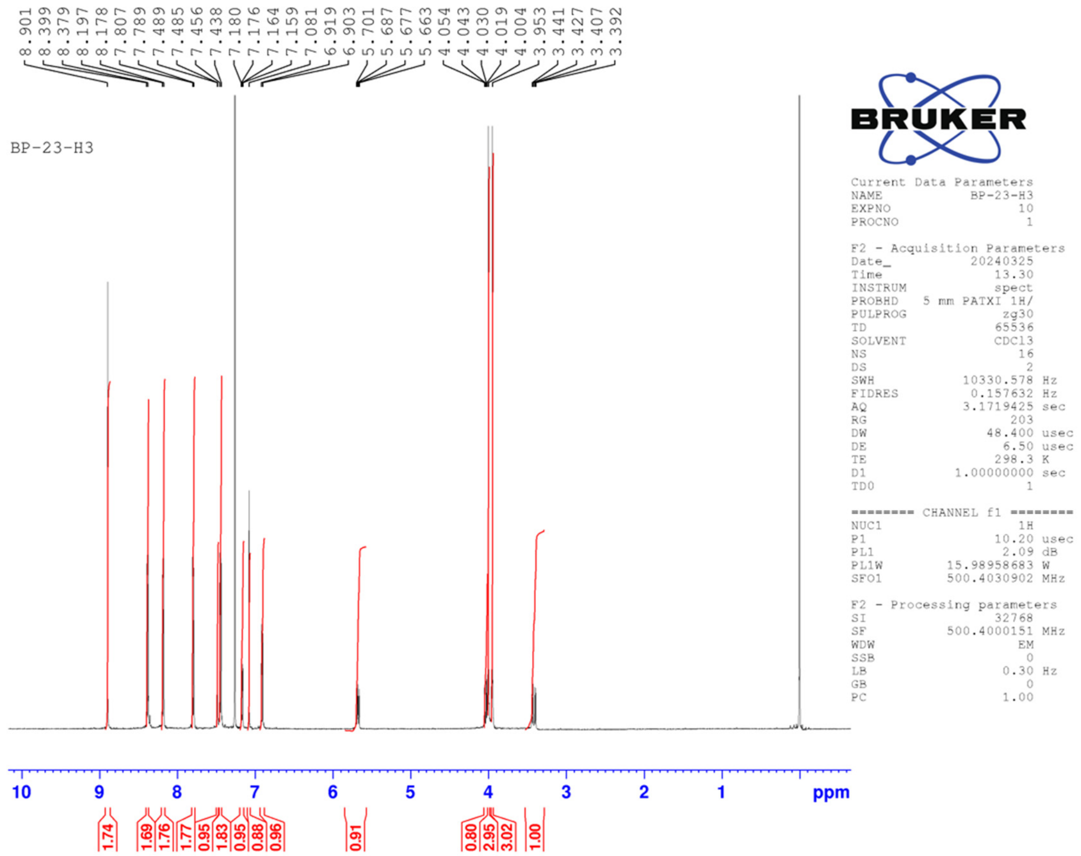

Figure S11: ^{1}H NMR Spectrum of **BH-2**

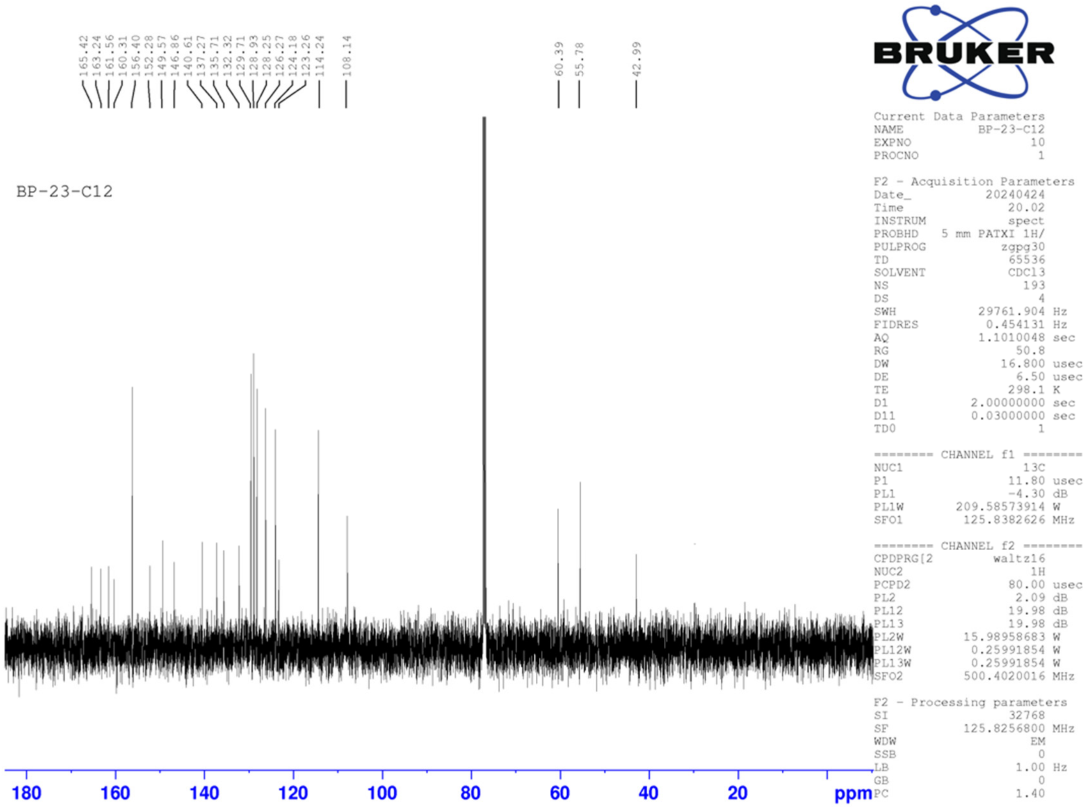

Figure S12: ^{13}C NMR Spectrum of **BH-2**

[Elemental Composition] Page: 1

Data : gousei996 Date : 27-Mar-2024 13:13

Sample: BP-23/sever belgin

Note : NBA

Inlet : Direct Ion Mode : FAB+

RT : 1.13 min Scan#: (3,8)

Elements : C 100/0, H 100/0, O 5/3, N 7/5, Cl 2/0, S 2/0

Mass Tolerance : 20ppm, 10mmu if m/z < 500, 20mmu if m/z > 1000

Unsaturation (U.S.) : -0.5 - 100.0

Observed m/z	Int%	Err[ppm / mmu]	U.S.	Composition
599.1232	12.6	+0.4 / +0.3	31.0	C 36 H 17 O 5 N 5
		-18.3 / -11.0	31.0	C 35 H 17 O 4 N 7
		-0.4 / -0.2	26.5	C 33 H 20 O 4 N 6 Cl
		+17.6 / +10.5	22.0	C 31 H 23 O 4 N 5 Cl 2
		-1.2 / -0.7	22.0	C 30 H 23 O 3 N 7 Cl 2
		-5.2 / -3.1	27.0	C 33 H 21 O 5 N 5 S
		+15.8 / +9.5	27.5	C 32 H 19 O 5 N 6 S
		-6.0 / -3.6	22.5	C 30 H 24 O 4 N 6 Cl S
		+15.0 / +9.0	23.0	C 29 H 22 O 4 N 7 Cl S
		+11.9 / +7.1	18.0	C 28 H 27 O 4 N 5 Cl 2 S
		-6.8 / -4.1	18.0	C 27 H 27 O 3 N 7 Cl 2 S
		-10.8 / -6.5	23.0	C 30 H 25 O 5 N 5 S 2
		+10.2 / +6.1	23.5	C 29 H 23 O 5 N 6 S 2
		-11.6 / -7.0	18.5	C 27 H 28 O 4 N 6 Cl S 2
		+9.4 / +5.6	19.0	C 26 H 26 O 4 N 7 Cl S 2
		+6.3 / +3.8	14.0	C 25 H 31 O 4 N 5 Cl 2 S 2
		-12.4 / -7.5	14.0	C 24 H 31 O 3 N 7 Cl 2 S 2

```
[ Theoretical Ion Distribution ]                                    Page: 1
Molecular Formula : C30 H24 O4 N6 Cl S
                    (m/z   599.1268, MW   600.0774, U.S. 22.5)
Base Peak :    599.1268, Averaged MW :   600.0772(a),   600.0794(w)

    m/z        INT.
  599.1268 100.0000 ***********************************************************
  600.1298  36.8728 *********************
  601.1252  43.8162 *************************
  602.1273  14.4513 ********
  603.1264   4.2420 **
  604.1268   0.9140 *
  605.1279   0.1533
  606.1292   0.0210
  607.1308   0.0024
  608.1325   0.0002
```

Figure S13: Mass Spectrum of **BH-2**

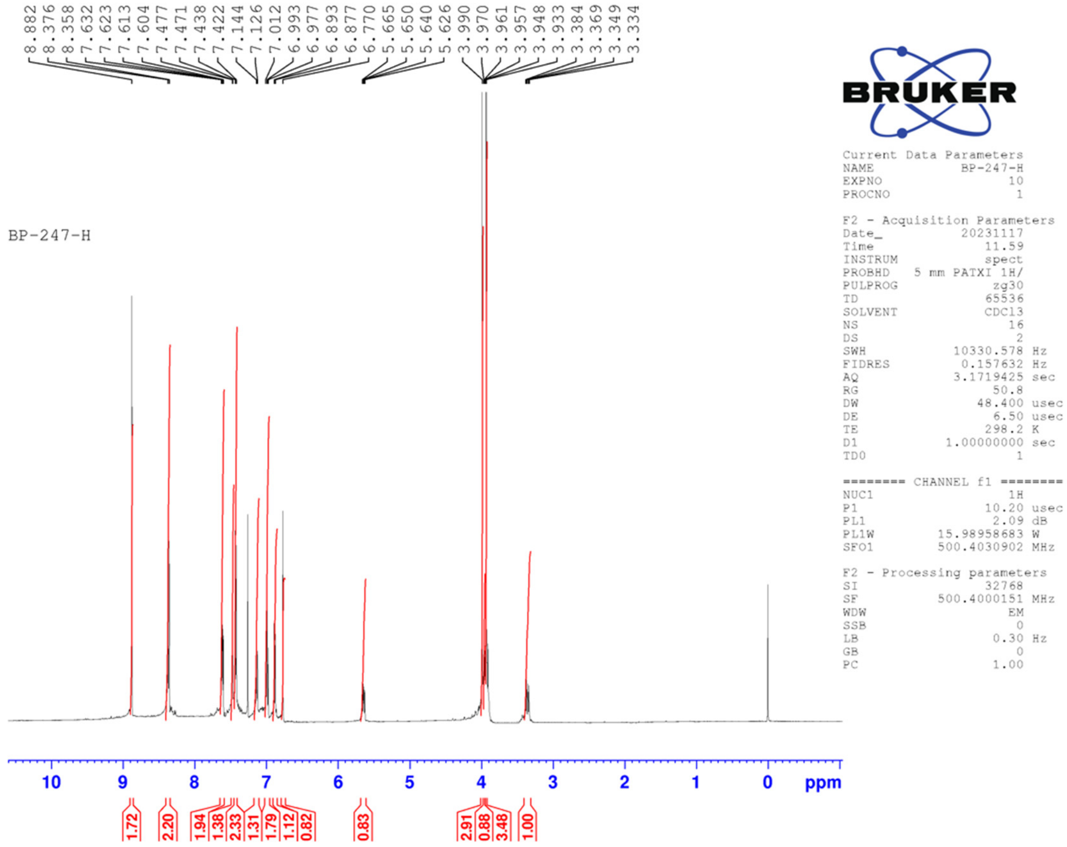

Figure S14: ¹H NMR Spectrum of **BH-3**

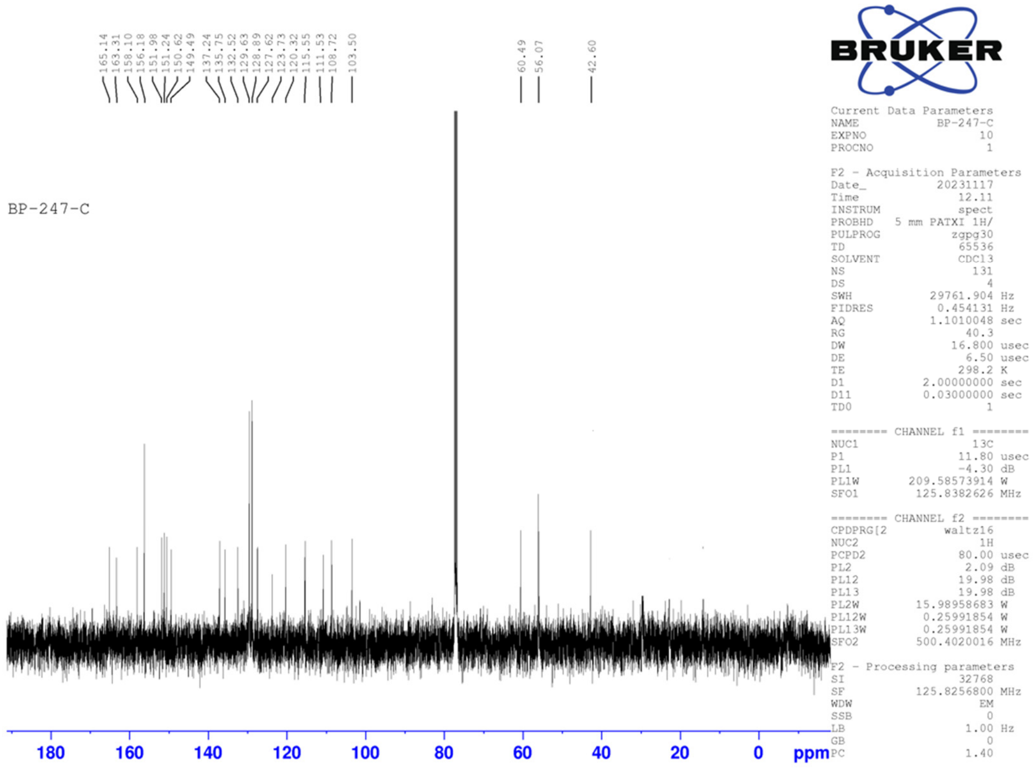

Figure S15: ^{13}C NMR Spectrum of **BH-3**

[Elemental Composition] Page: 1

Data : gousei801 Date : 21-Nov-2023 13:39

Sample: BP-24/sever belgin

Note : NBA

Inlet : Direct Ion Mode : FAB+

RT : 2.13 min Scan#: (8,11)

Elements : C 100/0, H 100/0, O 3/1, N 6/4, Cl 2/0, F 2/0, S 2/0

Mass Tolerance : 10ppm, 10mmu if m/z < 1000, 20mmu if m/z > 2000

Unsaturation (U.S.) : -0.5 - 100.0

Observed m/z	Int%	Err[ppm / mmu]	U.S.	Composition
571.1243	45.8	+8.5 / +4.8	34.5	C 39 H 15 O 2 N 4
		-11.2 / -6.4	34.5	C 38 H 15 O N 6
		-14.4 / -8.2	29.5	C 37 H 20 O N 4 Cl
		+7.6 / +4.3	30.0	C 36 H 18 O N 5 Cl
		-7.4 / -4.2	25.5	C 32 H 20 O 3 N 6 Cl
		-10.6 / -6.0	20.5	C 31 H 25 O 3 N 4 Cl 2
		+11.4 / +6.5	21.0	C 30 H 23 O 3 N 5 Cl 2
		+6.5 / +3.7	30.5	C 36 H 16 O 3 N 4 F
		-13.2 / -7.5	30.5	C 35 H 16 O 2 N 6 F
		-16.4 / -9.4	25.5	C 34 H 21 O 2 N 4 Cl F
		+5.6 / +3.2	26.0	C 33 H 19 O 2 N 5 Cl F
		-17.3 / -9.9	21.0	C 31 H 24 O N 5 Cl 2 F
		+4.7 / +2.7	21.5	C 30 H 22 O N 6 Cl 2 F
		-0.2 / -0.1	31.0	C 36 H 15 O N 5 F 2
		-15.2 / -8.7	26.5	C 32 H 17 O 3 N 6 F 2
		+3.6 / +2.1	22.0	C 30 H 20 O 3 N 5 Cl F 2
		+2.7 / +1.6	17.5	C 27 H 23 O 2 N 6 Cl 2 F 2
		+2.5 / +1.5	30.5	C 36 H 19 O 2 N 4 S
		-17.1 / -9.8	30.5	C 35 H 19 O N 6 S
		+1.7 / +1.0	26.0	C 33 H 22 O N 5 Cl S
		-13.3 / -7.6	21.5	C 29 H 24 O 3 N 6 Cl S
		-16.5 / -9.4	16.5	C 28 H 29 O 3 N 4 Cl 2 S
		+5.5 / +3.2	17.0	C 27 H 27 O 3 N 5 Cl 2 S
		+0.5 / +0.3	26.5	C 33 H 20 O 3 N 4 F S
		-0.3 / -0.2	22.0	C 30 H 23 O 2 N 5 Cl F S
		-1.2 / -0.7	17.5	C 27 H 26 O N 6 Cl 2 F S
		-6.1 / -3.5	27.0	C 33 H 19 O N 5 F 2 S
		+15.9 / +9.1	27.5	C 32 H 17 O N 6 F 2 S
		+12.7 / +7.2	22.5	C 31 H 22 O N 4 Cl F 2 S
		-2.3 / -1.3	18.0	C 27 H 24 O 3 N 5 Cl F 2 S
		+16.5 / +9.4	13.5	C 25 H 27 O 3 N 4 Cl 2 F 2 S
		-3.2 / -1.8	13.5	C 24 H 27 O 2 N 6 Cl 2 F 2 S
		-3.4 / -1.9	26.5	C 33 H 23 O 2 N 4 S 2
		-4.2 / -2.4	22.0	C 30 H 26 O N 5 Cl S 2
		+14.6 / +8.3	17.5	C 28 H 29 O N 4 Cl 2 S 2
		-0.4 / -0.2	13.0	C 24 H 31 O 3 N 5 Cl 2 S 2
		-5.4 / -3.1	22.5	C 30 H 24 O 3 N 4 F S 2
		+16.7 / +9.5	23.0	C 29 H 22 O 3 N 5 F S 2
		-6.2 / -3.5	18.0	C 27 H 27 O 2 N 5 Cl F S 2
		+15.8 / +9.0	18.5	C 26 H 25 O 2 N 6 Cl F S 2
		+12.6 / +7.2	13.5	C 25 H 30 O 2 N 4 Cl 2 F S 2
		-7.1 / -4.0	13.5	C 24 H 30 O N 6 Cl 2 F S 2
		-12.0 / -6.9	23.0	C 30 H 23 O N 5 F 2 S 2
		+10.0 / +5.7	23.5	C 29 H 21 O N 6 F 2 S 2
		+6.8 / +3.9	18.5	C 28 H 26 O N 4 Cl F 2 S 2
		-8.2 / -4.7	14.0	C 24 H 28 O 3 N 5 Cl F 2 S 2
		+13.8 / +7.9	14.5	C 23 H 26 O 3 N 6 Cl F 2 S 2
		+10.6 / +6.1	9.5	C 22 H 31 O 3 N 4 Cl 2 F 2 S 2
		-9.1 / -5.2	9.5	C 21 H 31 O 2 N 6 Cl 2 F 2 S 2
572.1276	51.2	+0.5 / +0.3	34.0	C 39 H 16 O 2 N 4
		-0.3 / -0.2	29.5	C 36 H 19 O N 5 Cl
		-15.3 / -8.7	25.0	C 32 H 21 O 3 N 6 Cl
		+3.5 / +2.0	20.5	C 30 H 24 O 3 N 5 Cl 2
		-1.5 / -0.8	30.0	C 36 H 17 O 3 N 4 F

```
[ Theoretical Ion Distribution ]                                  Page: 1
Molecular Formula : C30 H24 O2 N5 Cl F S
                     (m/z   572.1323, MW   573.0703, U.S. 21.5)
Base Peak :   572.1323, Averaged MW :   573.0703(a),   573.0725(w)

   m/z        INT.
 572.1323 100.0000 **********************************************************
 573.1353  36.4293 *********************
 574.1306  43.2533 *************************
 575.1328  14.1117 ********
 576.1317   4.0049 **
 577.1321   0.8387
 578.1332   0.1331
 579.1345   0.0169
 580.1361   0.0018
 581.1379   0.0002
```

Figure S16: Mass Spectrum of **BH-3**

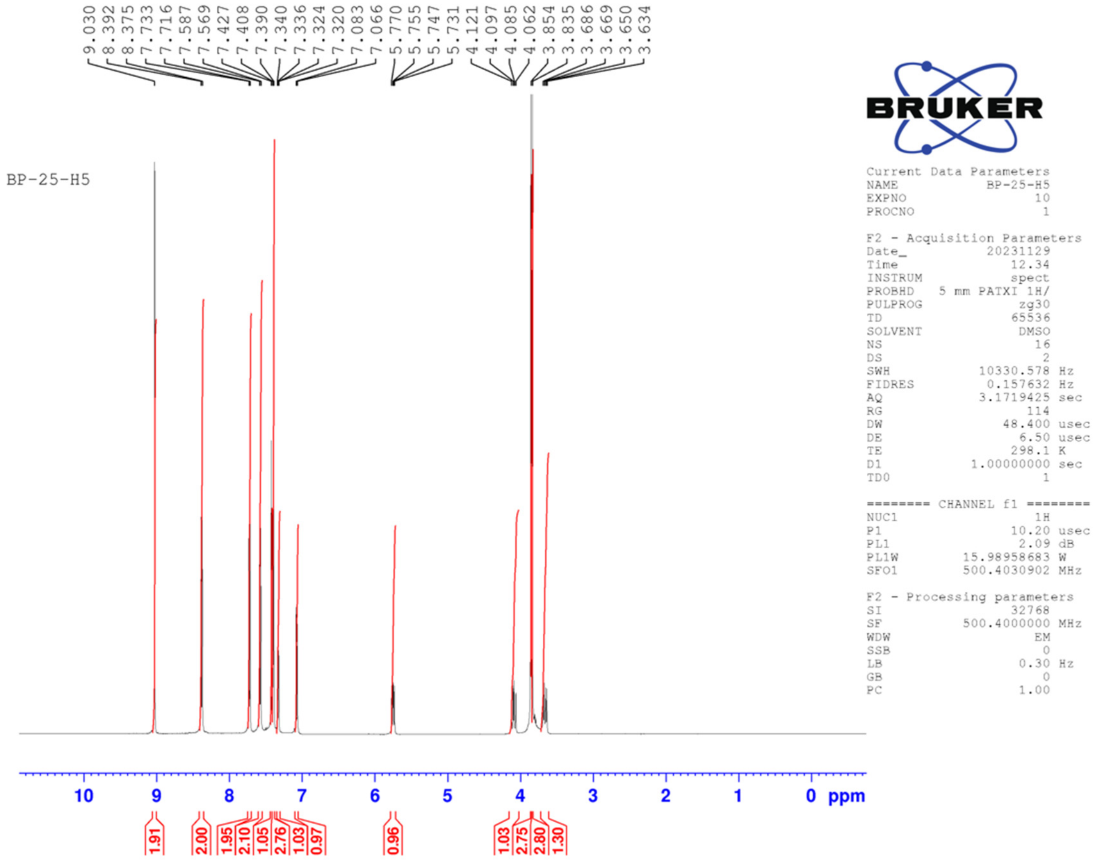

Figure S17: ^{1}H NMR Spectrum of **BH-4**

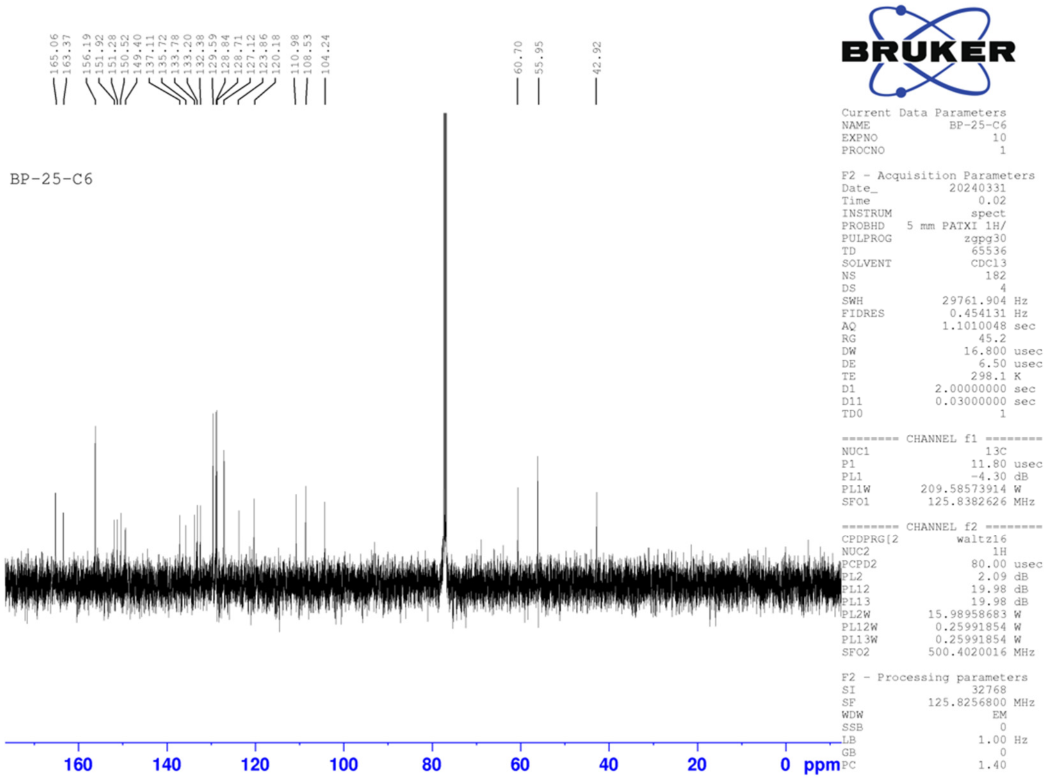

Figure S18: ^{13}C NMR Spectrum of **BH-4**

```
[ Elemental Composition ]                                          Page: 1
Data   : gousei803                    Date : 21-Nov-2023 14:21
Sample: BP-25/sever belgin
Note : NBA
Inlet : Direct                        Ion Mode : FAB+
RT : 1.50 min                         Scan#: (4,10)
Elements : C 100/0, H 100/0, O 3/1, N 6/4, Cl 3/1, S 2/0
Mass Tolerance      : 10ppm, 10mmu if m/z < 1000, 20mmu if m/z > 2000
Unsaturation (U.S.) : -0.5 - 100.0

Observed m/z  Int%   Err[ppm / mmu]    U.S.   Composition
  588.0955    55.0      -5.8 /  -3.4   30.0   C 36 H 17 O 3 N 4 Cl
                       +15.6 /  +9.2   30.5   C 35 H 15 O 3 N 5 Cl
                        -6.6 /  -3.9   25.5   C 33 H 20 O 2 N 5 Cl 2
                       +14.8 /  +8.7   26.0   C 32 H 18 O 2 N 6 Cl 2
                       +11.7 /  +6.9   21.0   C 31 H 23 O 2 N 4 Cl 3
                        -7.4 /  -4.4   21.0   C 30 H 23 O N 6 Cl 3
                       -11.5 /  -6.8   26.0   C 33 H 21 O 3 N 4 Cl S
                        +9.9 /  +5.8   26.5   C 32 H 19 O 3 N 5 Cl S
                       -12.3 /  -7.3   21.5   C 30 H 24 O 2 N 5 Cl 2 S
                        +9.0 /  +5.3   22.0   C 29 H 22 O 2 N 6 Cl 2 S
                        +5.9 /  +3.5   17.0   C 28 H 27 O 2 N 4 Cl 3 S
                       -13.2 /  -7.7   17.0   C 27 H 27 O N 6 Cl 3 S
                        +4.1 /  +2.4   22.5   C 29 H 23 O 3 N 5 Cl S 2
                        +3.3 /  +1.9   18.0   C 26 H 26 O 2 N 6 Cl 2 S 2
                        +0.2 /  +0.1   13.0   C 25 H 31 O 2 N 4 Cl 3 S 2

  587.0917    43.6      +1.1 /  +0.6   30.5   C 36 H 16 O 3 N 4 Cl
                        +0.3 /  +0.2   26.0   C 33 H 19 O 2 N 5 Cl 2
                        -0.6 /  -0.3   21.5   C 30 H 22 O N 6 Cl 3
                        -4.6 /  -2.7   26.5   C 33 H 20 O 3 N 4 Cl S
                       +16.8 /  +9.8   27.0   C 32 H 18 O 3 N 5 Cl S
                        -5.5 /  -3.2   22.0   C 30 H 23 O 2 N 5 Cl 2 S
                       +15.9 /  +9.4   22.5   C 29 H 21 O 2 N 6 Cl 2 S
                       +12.8 /  +7.5   17.5   C 28 H 26 O 2 N 4 Cl 3 S
                        -6.3 /  -3.7   17.5   C 27 H 26 O N 6 Cl 3 S
                       -10.4 /  -6.1   22.5   C 30 H 24 O 3 N 4 Cl S 2
                       +11.0 /  +6.5   23.0   C 29 H 22 O 3 N 5 Cl S 2
                       -11.2 /  -6.6   18.0   C 27 H 27 O 2 N 5 Cl 2 S 2
                       +10.2 /  +6.0   18.5   C 26 H 25 O 2 N 6 Cl 2 S 2
                        +7.1 /  +4.2   13.5   C 25 H 30 O 2 N 4 Cl 3 S 2
                       -12.1 /  -7.1   13.5   C 24 H 30 O N 6 Cl 3 S 2
```

```
[ Theoretical Ion Distribution ]                                   Page: 1
Molecular Formula : C30 H24 O2 N5 Cl2 S
                         (m/z   588.1028, MW   589.5249, U.S. 21.5)
Base Peak :   588.1028, Averaged MW :   589.5246(a),   589.5280(w)

   m/z       INT.
 588.1028 100.0000 ************************************************************
 589.1058  36.4293 **********************
 590.1006  75.2317 *********************************************
 591.1030  25.7612 ***************
 592.0990  17.8366 **********
 593.1006   5.3514 ***
 594.0996   1.4138 *
 595.0999   0.2851
 596.1009   0.0444
 597.1022   0.0056
 598.1037   0.0006
```

Figure S19: Mass Spectrum of **BH-4**

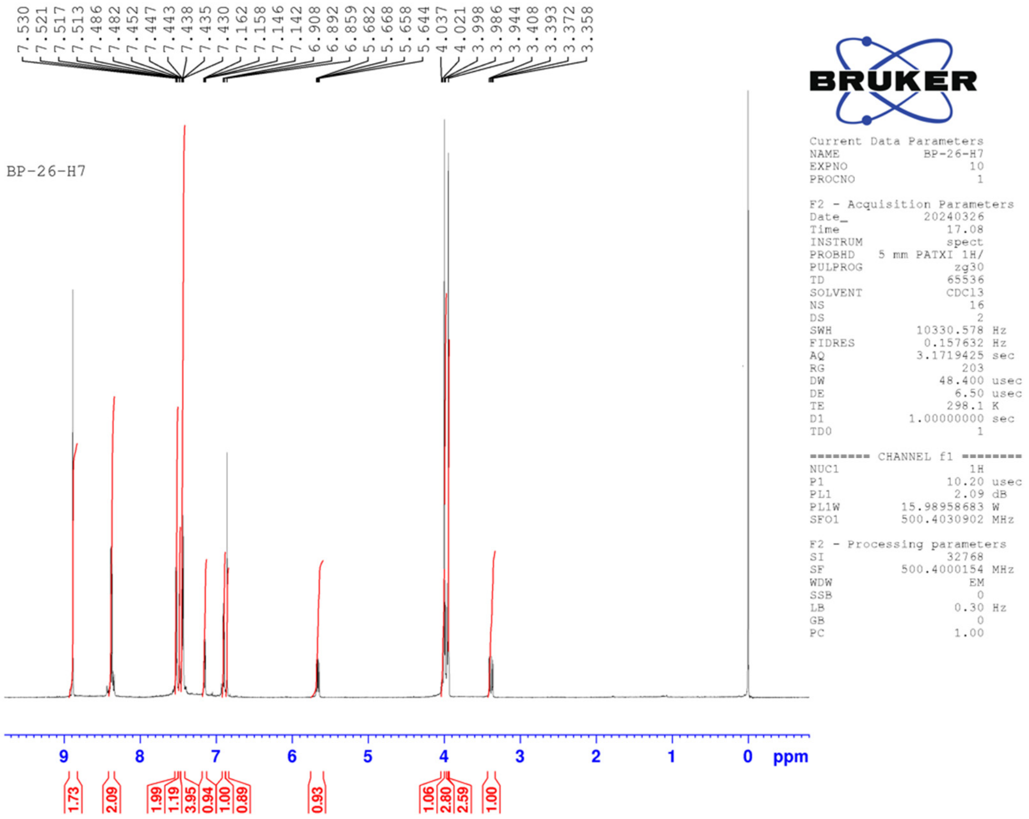

Figure S20: ^{1}H NMR Spectrum of **BH-5**

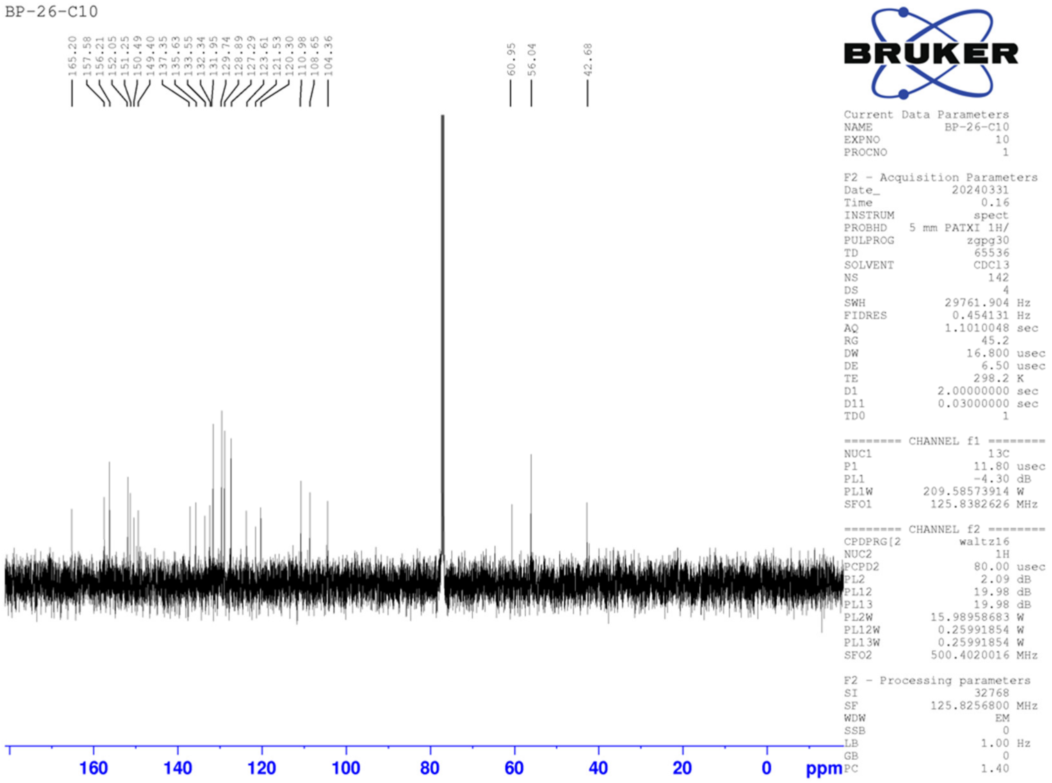

Figure S21: ^{13}C NMR Spectrum of **BH-5**

[Elemental Composition] Page: 1

Data : gousei997 Date : 27-Mar-2024 13:33
Sample: BP-26/sever belgin
Note : NBA
Inlet : Direct Ion Mode : FAB+
RT : 1.50 min Scan#: 7
Elements : C 100/0, H 100/0, O 3/1, N 6/4, Cl 2/0, Br 2/0, S 2/0
Mass Tolerance : 20ppm, 10mmu if m/z < 500, 20mmu if m/z > 1000
Unsaturation (U.S.) : -0.5 - 100.0

Observed m/z	Int%	Err[ppm / mmu]	U.S.	Composition
632.0474	35.8	-15.5 / -9.8	45.5	C 45 H 6 O N 5
		+4.4 / +2.8	46.0	C 44 H 4 O N 6
		+1.5 / +0.9	41.0	C 43 H 9 O N 4 Cl
		-12.1 / -7.6	36.5	C 39 H 11 O 3 N 5 Cl
		+7.8 / +5.0	37.0	C 38 H 9 O 3 N 6 Cl
		+4.9 / +3.1	32.0	C 37 H 14 O 3 N 4 Cl 2
		-12.8 / -8.1	32.0	C 36 H 14 O 2 N 6 Cl 2
		-1.5 / -1.0	30.0	C 36 H 17 O 3 N 4 Br
		+18.4 / +11.6	30.5	C 35 H 15 O 3 N 5 Br
		-19.3 / -12.2	30.0	C 35 H 17 O 2 N 6 Br
		-2.3 / -1.5	25.5	C 33 H 20 O 2 N 5 Cl Br
		+17.6 / +11.1	26.0	C 32 H 18 O 2 N 6 Cl Br
		+14.7 / +9.3	21.0	C 31 H 23 O 2 N 4 Cl 2 Br
		-3.1 / -2.0	21.0	C 30 H 23 O N 6 Cl 2 Br
		+8.2 / +5.2	19.0	C 30 H 26 O 2 N 4 Br 2
		-9.6 / -6.1	19.0	C 29 H 26 O N 6 Br 2
		-12.5 / -7.9	14.0	C 28 H 31 O N 4 Cl Br 2
		+7.4 / +4.7	14.5	C 27 H 29 O N 5 Cl Br 2
		-6.1 / -3.9	10.0	C 23 H 31 O 3 N 6 Cl Br 2
		-9.0 / -5.7	5.0	C 22 H 36 O 3 N 4 Cl 2 Br 2
		+10.9 / +6.9	5.5	C 21 H 34 O 3 N 5 Cl 2 Br 2
		+16.8 / +10.6	42.0	C 42 H 8 O 2 N 4 S
		-1.0 / -0.6	42.0	C 41 H 8 O N 6 S
		-3.9 / -2.4	37.0	C 40 H 13 O N 4 Cl S
		+16.0 / +10.1	37.5	C 39 H 11 O N 5 Cl S
		-17.4 / -11.0	32.5	C 36 H 15 O 3 N 5 Cl S
		+2.5 / +1.6	33.0	C 35 H 13 O 3 N 6 Cl S
		-0.4 / -0.2	28.0	C 34 H 18 O 3 N 4 Cl 2 S
		+19.5 / +12.3	28.5	C 33 H 16 O 3 N 5 Cl 2 S
		-18.2 / -11.5	28.0	C 33 H 18 O 2 N 6 Cl 2 S
		-6.9 / -4.3	26.0	C 33 H 21 O 3 N 4 Br S
		+13.0 / +8.2	26.5	C 32 H 19 O 3 N 5 Br S
		-7.7 / -4.8	21.5	C 30 H 24 O 2 N 5 Cl Br S
		+12.2 / +7.7	22.0	C 29 H 22 O 2 N 6 Cl Br S
		+9.4 / +5.9	17.0	C 28 H 27 O 2 N 4 Cl 2 Br S
		-8.4 / -5.3	17.0	C 27 H 27 O N 6 Cl 2 Br S
		+2.9 / +1.8	15.0	C 27 H 30 O 2 N 4 Br 2 S
		-14.9 / -9.4	15.0	C 26 H 30 O N 6 Br 2 S
		-17.8 / -11.3	10.0	C 25 H 35 O N 4 Cl Br 2 S
		+2.1 / +1.3	10.5	C 24 H 33 O N 5 Cl Br 2 S
		-11.4 / -7.2	6.0	C 20 H 35 O 3 N 6 Cl Br 2 S
		+19.1 / +12.1	6.0	C 22 H 36 O N 4 Cl 2 Br 2 S
		-14.3 / -9.1	1.0	C 19 H 40 O 3 N 4 Cl 2 Br 2 S
		+5.6 / +3.5	1.5	C 18 H 38 O 3 N 5 Cl 2 Br 2 S
		+11.5 / +7.3	38.0	C 39 H 12 O 2 N 4 S 2
		-6.3 / -4.0	38.0	C 38 H 12 O N 6 S 2
		-9.2 / -5.8	33.0	C 37 H 17 O N 4 Cl S 2
		+10.7 / +6.8	33.5	C 36 H 15 O N 5 Cl S 2
		-2.8 / -1.8	29.0	C 32 H 17 O 3 N 6 Cl S 2
		-5.7 / -3.6	24.0	C 31 H 22 O 3 N 4 Cl 2 S 2
		+14.2 / +9.0	24.5	C 30 H 20 O 3 N 5 Cl 2 S 2
		-12.2 / -7.7	22.0	C 30 H 25 O 3 N 4 Br S 2
		+7.7 / +4.9	22.5	C 29 H 23 O 3 N 5 Br S 2
		-13.0 / -8.2	17.5	C 27 H 28 O 2 N 5 Cl Br S 2
		+6.9 / +4.4	18.0	C 26 H 26 O 2 N 6 Cl Br S 2
		+4.0 / +2.5	13.0	C 25 H 31 O 2 N 4 Cl 2 Br S 2
		-13.8 / -8.7	13.0	C 24 H 31 O N 6 Cl 2 Br S 2
		-2.5 / -1.6	11.0	C 24 H 34 O 2 N 4 Br 2 S 2
		+17.4 / +11.0	11.5	C 23 H 32 O 2 N 5 Br 2 S 2
		-3.3 / -2.1	6.5	C 21 H 37 O N 5 Cl Br 2 S 2
		+16.6 / +10.5	7.0	C 20 H 35 O N 6 Cl Br 2 S 2
		-16.8 / -10.6	2.0	C 17 H 39 O 3 N 6 Cl Br 2 S 2
		+13.8 / +8.7	2.0	C 19 H 40 O N 4 Cl 2 Br 2 S 2

[Elemental Composition] Page: 2

```
[ Theoretical Ion Distribution ]                                    Page: 1
Molecular Formula : C30 H24 O2 N5 Cl Br S
                    (m/z    632.0523, MW    633.9759, U.S. 21.5)
Base Peak :    634.0503, Averaged MW :    633.9754(a),    633.9790(w)

    m/z        INT.
  632.0523  71.1587 *****************************************
  633.0552  25.9226 ***************
  634.0503 100.0000 **********************************************************
  635.0531  35.2586 ********************
  636.0488  32.7904 *******************
  637.0507  10.3651 ******
  638.0497   2.8670 **
  639.0501   0.5926
  640.0511   0.0934
  641.0525   0.0118
  642.0540   0.0012
  643.0558   0.0001
```

Figure S22: Mass Spectrum of **BH-5**

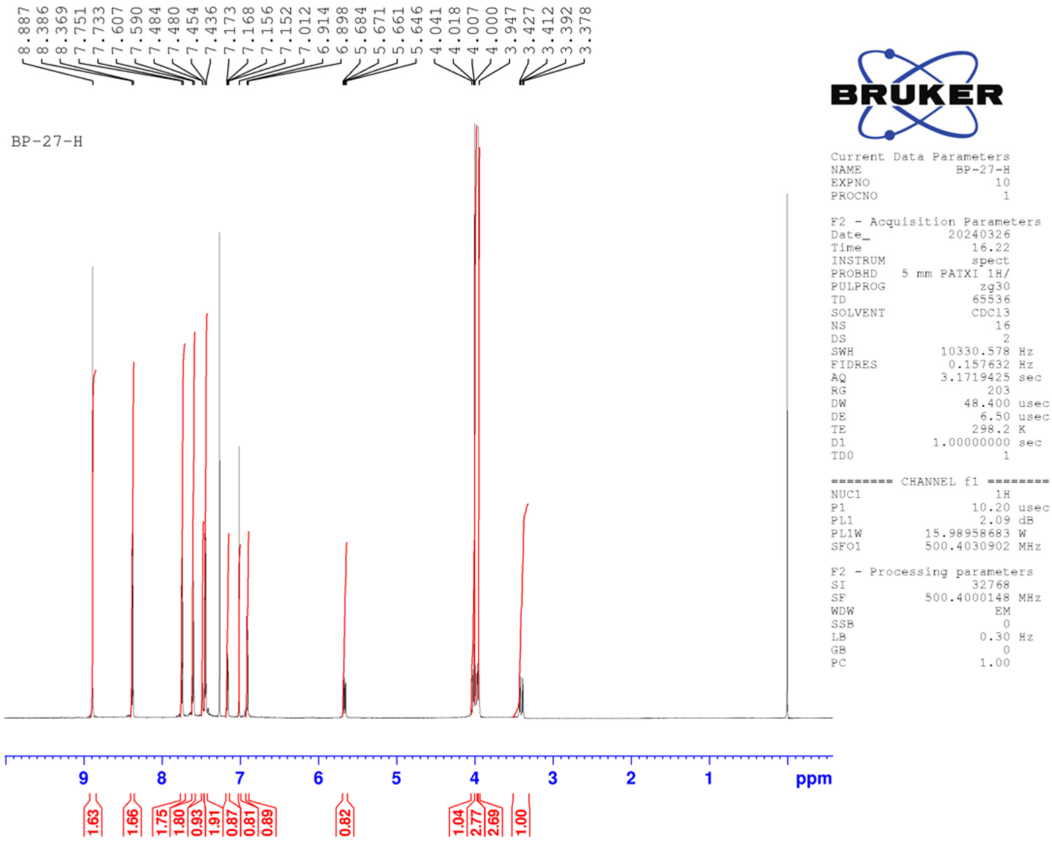

Figure S23: ^{1}H NMR Spectrum of **BH-6**

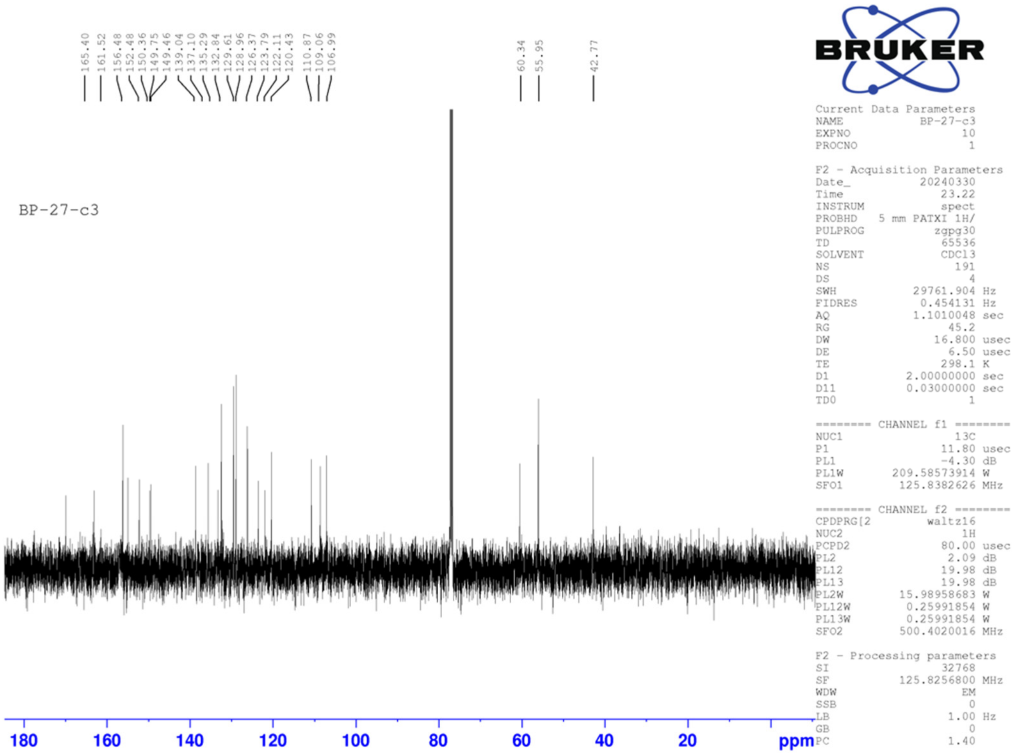

Figure S24: ^{13}C NMR Spectrum of **BH-6**

[Elemental Composition] Page: 1

Data : gousei998 Date : 27-Mar-2024 13:48

Sample: BP-27/sever belgin

Note : NBA

Inlet : Direct Ion Mode : FAB+

RT : 1.13 min Scan#: (3,8)

Elements : C 100/0, H 100/0, O 3/1, N 7/5, Cl 2/0, S 2/0

Mass Tolerance : 20ppm, 10mmu if m/z < 500, 20mmu if m/z > 1000

Unsaturation (U.S.) : -0.5 - 100.0

Observed m/z	Int%	Err[ppm / mmu]	U.S.	Composition
578.1288	59.2	+6.1 / +3.5	32.5	C 37 H 16 O 3 N 5
		-13.3 / -7.7	32.5	C 36 H 16 O 2 N 7
		-16.5 / -9.5	27.5	C 35 H 21 O 2 N 5 Cl
		+5.3 / +3.0	28.0	C 34 H 19 O 2 N 6 Cl
		-17.3 / -10.0	23.0	C 32 H 24 O N 6 Cl 2
		+4.4 / +2.6	23.5	C 31 H 22 O N 7 Cl 2
		+0.3 / +0.2	28.5	C 34 H 20 O 3 N 5 S
		-19.2 / -11.1	28.5	C 33 H 20 O 2 N 7 S
		-0.6 / -0.3	24.0	C 31 H 23 O 2 N 6 Cl S
		+18.0 / +10.4	19.5	C 29 H 26 O 2 N 5 Cl 2 S
		-1.4 / -0.8	19.5	C 28 H 26 O N 7 Cl 2 S
		-5.6 / -3.2	24.5	C 31 H 24 O 3 N 5 S 2
		+16.2 / +9.4	25.0	C 30 H 22 O 3 N 6 S 2
		-6.4 / -3.7	20.0	C 28 H 27 O 2 N 6 Cl S 2
		+15.4 / +8.9	20.5	C 27 H 25 O 2 N 7 Cl S 2
		+12.2 / +7.0	15.5	C 26 H 30 O 2 N 5 Cl 2 S 2
		-7.2 / -4.2	15.5	C 25 H 30 O N 7 Cl 2 S 2
579.1351	53.6	+3.4 / +2.0	32.0	C 37 H 17 O 3 N 5
		-16.0 / -9.2	32.0	C 36 H 17 O 2 N 7
		-19.1 / -11.1	27.0	C 35 H 22 O 2 N 5 Cl
		+2.6 / +1.5	27.5	C 34 H 20 O 2 N 6 Cl
		-20.0 / -11.6	22.5	C 32 H 25 O N 6 Cl 2
		+1.8 / +1.0	23.0	C 31 H 23 O N 7 Cl 2
		-2.4 / -1.4	28.0	C 34 H 21 O 3 N 5 S
		+19.3 / +11.2	28.5	C 33 H 19 O 3 N 6 S
		-3.2 / -1.9	23.5	C 31 H 24 O 2 N 6 Cl S
		+18.5 / +10.7	24.0	C 30 H 22 O 2 N 7 Cl S
		+15.3 / +8.9	19.0	C 29 H 27 O 2 N 5 Cl 2 S
		-4.1 / -2.4	19.0	C 28 H 27 O N 7 Cl 2 S
		-8.2 / -4.8	24.0	C 31 H 25 O 3 N 5 S 2
		+13.5 / +7.8	24.5	C 30 H 23 O 3 N 6 S 2
		-9.0 / -5.2	19.5	C 28 H 28 O 2 N 6 Cl S 2
		+12.7 / +7.3	20.0	C 27 H 26 O 2 N 7 Cl S 2
		+9.5 / +5.5	15.0	C 26 H 31 O 2 N 5 Cl 2 S 2
		-9.9 / -5.7	15.0	C 25 H 31 O N 7 Cl 2 S 2

[Theoretical Ion Distribution] Page: 1

Molecular Formula : C31 H23 O2 N6 Cl S

(m/z 578.1292, MW 579.0816, U.S. 24.0)

Base Peak : 578.1292, Averaged MW : 579.0817(a), 579.0839(w)

m/z	INT.	
578.1292	100.0000	**
579.1321	37.8939	**********************
580.1276	43.7907	*************************
581.1296	14.7465	*********
582.1287	4.2132	**
583.1290	0.8979	*
584.1301	0.1456	
585.1314	0.0189	
586.1329	0.0020	
587.1347	0.0002	

Figure S25: Mass Spectrum of **BH-6**

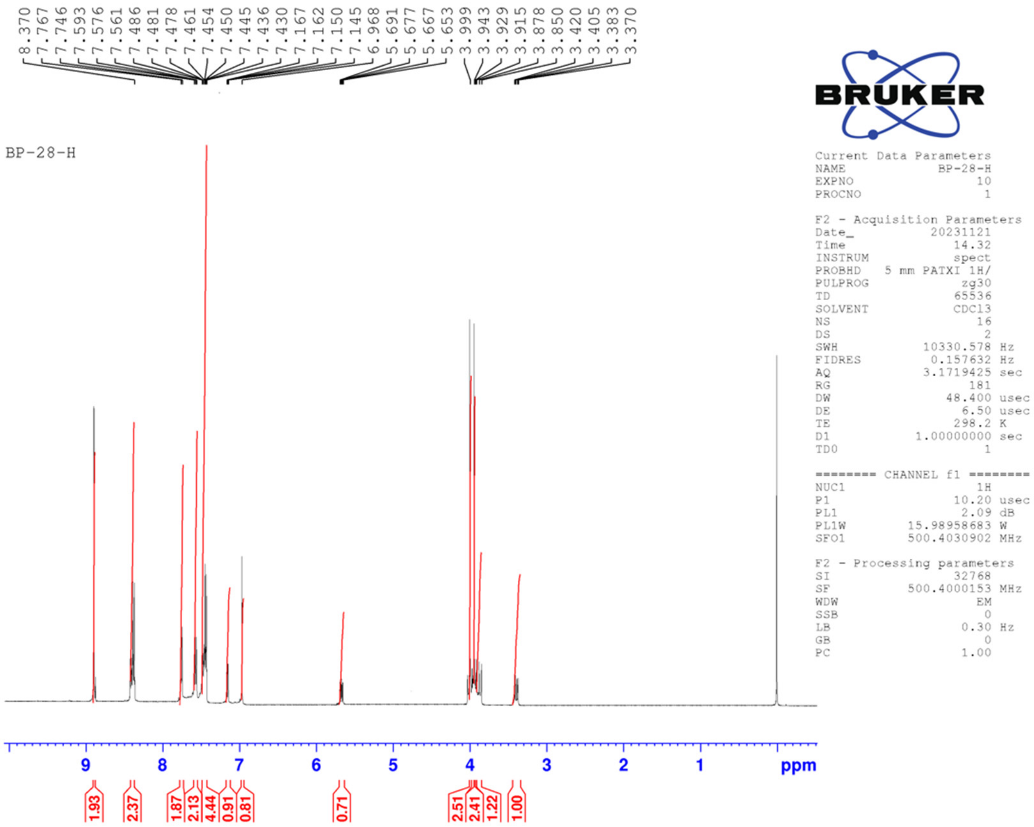

Figure S26: ^{1}H NMR Spectrum of **BH-7**

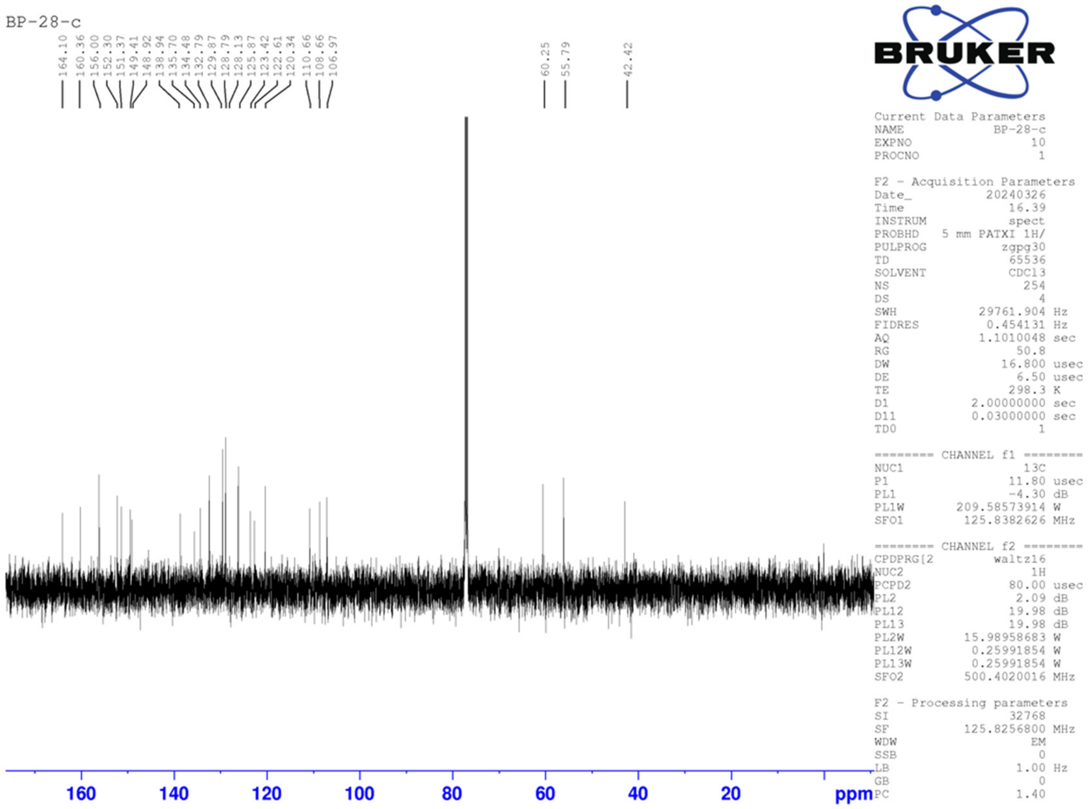

Figure S27: ^{13}C NMR Spectrum of **BH-7**

[Elemental Composition] Page: 1
Data : gousei808 Date : 22-Nov-2023 09:37
Sample: BP-28/sever belgin
Note : NBA
Inlet : Direct Ion Mode : FAB+
RT : 1.00 min Scan#: (3,7)
Elements : C 100/0, H 100/0, O 3/1, N 6/4, Cl 2/0, F 4/2, S 2/0
Mass Tolerance : 10ppm, 10mmu if m/z < 1000, 20mmu if m/z > 2000
Unsaturation (U.S.) : -0.5 - 100.0

Observed m/z	Int%	Err[ppm / mmu]	U.S.	Composition
621.1205	100.0	+6.8 / +4.2	34.5	C 40 H 15 O 2 N 4 F 2
		-11.3 / -7.0	34.5	C 39 H 15 O N 6 F 2
		-14.2 / -8.8	29.5	C 38 H 20 O N 4 Cl F 2
		+6.0 / +3.7	30.0	C 37 H 18 O N 5 Cl F 2
		-7.7 / -4.8	25.5	C 33 H 20 O 3 N 6 Cl F 2
		-10.7 / -6.6	20.5	C 32 H 25 O 3 N 4 Cl 2 F 2
		+9.6 / +5.9	21.0	C 31 H 23 O 3 N 5 Cl 2 F 2
		+5.0 / +3.1	30.5	C 37 H 16 O 3 N 4 F 3
		-13.1 / -8.1	30.5	C 36 H 16 O 2 N 6 F 3
		-16.1 / -10.0	25.5	C 35 H 21 O 2 N 4 Cl F 3
		+4.2 / +2.6	26.0	C 34 H 19 O 2 N 5 Cl F 3
		+3.4 / +2.1	21.5	C 31 H 22 O N 6 Cl 2 F 3
		-1.2 / -0.7	31.0	C 37 H 15 O N 5 F 4
		-14.9 / -9.3	26.5	C 33 H 17 O 3 N 6 F 4
		+2.4 / +1.5	22.0	C 31 H 20 O 3 N 5 Cl F 4
		+1.6 / +1.0	17.5	C 28 H 23 O 2 N 6 Cl 2 F 4
		+1.4 / +0.9	30.5	C 37 H 19 O 2 N 4 F 2 S
		+0.6 / +0.4	26.0	C 34 H 22 O N 5 Cl F 2 S
		-13.2 / -8.2	21.5	C 30 H 24 O 3 N 6 Cl F 2 S
		+4.1 / +2.6	17.0	C 28 H 27 O 3 N 5 Cl 2 F 2 S
		-0.4 / -0.3	26.5	C 34 H 20 O 3 N 4 F 3 S
		-1.2 / -0.8	22.0	C 31 H 23 O 2 N 5 Cl F 3 S
		+16.1 / +10.0	17.5	C 29 H 26 O 2 N 4 Cl 2 F 3 S
		-2.0 / -1.3	17.5	C 28 H 26 O N 6 Cl 2 F 3 S
		-6.6 / -4.1	27.0	C 34 H 19 O N 5 F 4 S
		+13.6 / +8.5	27.5	C 33 H 17 O N 6 F 4 S
		+10.7 / +6.6	22.5	C 32 H 22 O N 4 Cl F 4 S
		-3.1 / -1.9	18.0	C 28 H 24 O 3 N 5 Cl F 4 S
		+14.2 / +8.8	13.5	C 26 H 27 O 3 N 4 Cl 2 F 4 S
		-3.9 / -2.4	13.5	C 25 H 27 O 2 N 6 Cl 2 F 4 S
		-4.0 / -2.5	26.5	C 34 H 23 O 2 N 4 F 2 S 2
		-4.8 / -3.0	22.0	C 31 H 26 O N 5 Cl F 2 S 2
		+15.4 / +9.6	22.5	C 30 H 24 O N 6 Cl F 2 S 2
		+12.5 / +7.8	17.5	C 29 H 29 O N 4 Cl 2 F 2 S 2
		-1.3 / -0.8	13.0	C 25 H 31 O 3 N 5 Cl 2 F 2 S 2
		-5.9 / -3.6	22.5	C 31 H 24 O 3 N 4 F 3 S 2
		+14.4 / +8.9	23.0	C 30 H 22 O 3 N 5 F 3 S 2
		-6.7 / -4.1	18.0	C 28 H 27 O 2 N 5 Cl F 3 S 2
		+13.6 / +8.4	18.5	C 27 H 25 O 2 N 6 Cl F 3 S 2
		+10.6 / +6.6	13.5	C 26 H 30 O 2 N 4 Cl 2 F 3 S 2
		-7.4 / -4.6	13.5	C 25 H 30 O N 6 Cl 2 F 3 S 2
		-12.0 / -7.5	23.0	C 31 H 23 O N 5 F 4 S 2
		+8.2 / +5.1	23.5	C 30 H 21 O N 6 F 4 S 2
		+5.3 / +3.3	18.5	C 29 H 26 O N 4 Cl F 4 S 2
		-8.5 / -5.3	14.0	C 25 H 28 O 3 N 5 Cl F 4 S 2
		+11.7 / +7.3	14.5	C 24 H 26 O 3 N 6 Cl F 4 S 2
		+8.8 / +5.5	9.5	C 23 H 31 O 3 N 4 Cl 2 F 4 S 2
		-9.3 / -5.8	9.5	C 22 H 31 O 2 N 6 Cl 2 F 4 S 2

[Theoretical Ion Distribution] Page: 1
Molecular Formula : C31 H23 O2 N5 Cl F3 S
(m/z 621.1213, MW 622.0701, U.S. 22.0)
Base Peak : 621.1213, Averaged MW : 622.0702(a), 622.0722(w)

m/z	INT.	
621.1213	100.0000	**
622.1243	37.5265	**********************
623.1197	43.6529	*************************
624.1218	14.5862	********
625.1209	4.1597	**
626.1212	0.8827	*
627.1223	0.1423	
628.1237	0.0184	
629.1252	0.0020	
630.1270	0.0002	

Figure S28: Mass Spectrum of **BH-7**
